# Inverse design in quantum nanophotonics: combining local-density-of-states and deep learning

**DOI:** 10.1515/nanoph-2022-0746

**Published:** 2023-04-13

**Authors:** Guang-Xin Liu, Jing-Feng Liu, Wen-Jie Zhou, Ling-Yan Li, Chun-Lian You, Cheng-Wei Qiu, Lin Wu

**Affiliations:** College of Electronic Engineering and College of Artificial Intelligence, South China Agricultural University, Guangzhou 510642, China; Science, Mathematics and Technology (SMT), Singapore University of Technology and Design (SUTD), 8 Somapah Road, Singapore 487372, Singapore; Department of Electrical and Computer Engineering, National University of Singapore, 4 Engineering Drive 3, Singapore 117583, Singapore; Agency for Science, Technology, and Research (A*STAR), Institute of High Performance Computing, Singapore 138632, Singapore

**Keywords:** deep learning, entanglement dynamics, inverse design, local density of states, nanophotonics, spontaneous emission dynamics

## Abstract

Recent advances in inverse-design approaches for discovering optical structures based on desired functional characteristics have reshaped the landscape of nanophotonic structures, where most studies have focused on how light interacts with nanophotonic structures only. When quantum emitters (QEs), such as atoms, molecules, and quantum dots, are introduced to couple to the nanophotonic structures, the light–matter interactions become much more complicated, forming a rapidly developing field – quantum nanophotonics. Typical quantum functional characteristics depend on the intrinsic properties of the QE and its electromagnetic environment created by the nanophotonic structures, commonly represented by a scalar quantity, local-density-of-states (LDOS). In this work, we introduce a generalized inverse-design framework in quantum nanophotonics by taking LDOS as the bridge to connect the nanophotonic structures and the quantum functional characteristics. We take a simple system consisting of QEs sitting on a single multilayer shell–metal–nanoparticle (SMNP) as an example, apply fully-connected neural networks to model the LDOS of SMNP, inversely design and optimize the geometry of the SMNP based on LDOS, and realize desirable quantum characteristics in two quantum nanophotonic problems: spontaneous emission and entanglement. Our work introduces deep learning to the quantum optics domain for advancing quantum device designs; and provides a new platform for practicing deep learning to design nanophotonic structures for complex problems without a direct link between structures and functional characteristics.

## 1 Introduction

Quantum nanophotonics is a rapidly developing field exploring how light interacts with atoms, molecules, and artificial nanophotonic structures. In recent years, the designs of artificial nanophotonic structures have been tremendously expanded by inverse-design engineering [1], such as genetic algorithm [2–4], adjoint method [5], and deep learning (DL) [6, 7]. While most of these approaches demand immense computation time or highly depend on past design experiences [8], DL is a more efficient black-box algorithm for designing the nanophotonic structures and manipulating the functionalities, as it adopts neural networks (NNs) to reflect the function between nanophotonic structures and their functionalities directly. For example, advanced artificial NNs have been employed for regulating the scatterings and absorptions in various nanophotonic structures such as core–shell nanoparticles [9], multilayer films [10], and metasurfaces [11, 12]. A simultaneous inverse design of materials and structure parameters of core–shell nanoparticles has been achieved using deep learning of a neural network for dipole resonance engineering [13]. Also, DL has been used to automatically design and optimize 3D chiral metamaterials with strong chiroptical responses at predesignated wavelengths via a partial stacking strategy [14], and a semi-supervised learning strategy [15]. Similar enormous NNs have been applied to design and optimize complicated chiral plasmonic metamaterials [16], multifunctional metasurfaces [17], holographic 3D particle imaging [18], and integrated photonic power splitters [19], where these adopted NNs are unavoidably tremendous because they directly learn large-scale and complex systems. Nevertheless, these pioneering studies have only focused on how light interacts with artificial nanophotonic structures.

In quantum nanophotonics, quantum emitters (QEs), such as atoms, molecules, and quantum dots, are introduced to couple to artificial nanophotonic structures and light–matter interactions have become much more complicated [20]. Engineering such interactions give rise to novel quantum applications, such as quantum cascade lasers [21], quantum-optical microcombs [22], and quantum-optical networks [23, 24]. Direct application of DL techniques to such quantum nanophotonic problems may require even larger NN structures with lengthy calculations and adjustments. An efficient solution could be taking an intermediate quantity to simplify the inverse-design problems. Generally, in quantum nanophotonic problems, the quantum characteristics (e.g., spontaneous emission from QEs [25, 26], qubit entanglement dynamics [27, 28]) depend on the intrinsic properties of the QE and can also be manipulated by designing the artificial nanophotonic structures, which principally determine the electromagnetic (EM) environment [29, 30] around the QEs. Such an EM environment is often characterized by a scalar quantity, the local-density-of-states (LDOS), representing the number of EM field modes per unit volume and frequency at a given point in space [31]. In principle, LDOS can fully describe the interactions of a quantum emitter with an arbitrary EM environment [32, 33], and LDOS has been taken as an intermediate quantity to enhance the spontaneous emission from a QE in a photonic crystal structure with topology optimization [34]. In this sense, LDOS can be considered the “bridge” between the quantum characteristics and the artificial nanophotonic structures. Combining LDOS and deep learning, nanophotonic structures can be inversely designed and optimized to obtain the desired quantum characteristics for various quantum nanophotonic applications.

**Figure 1:**
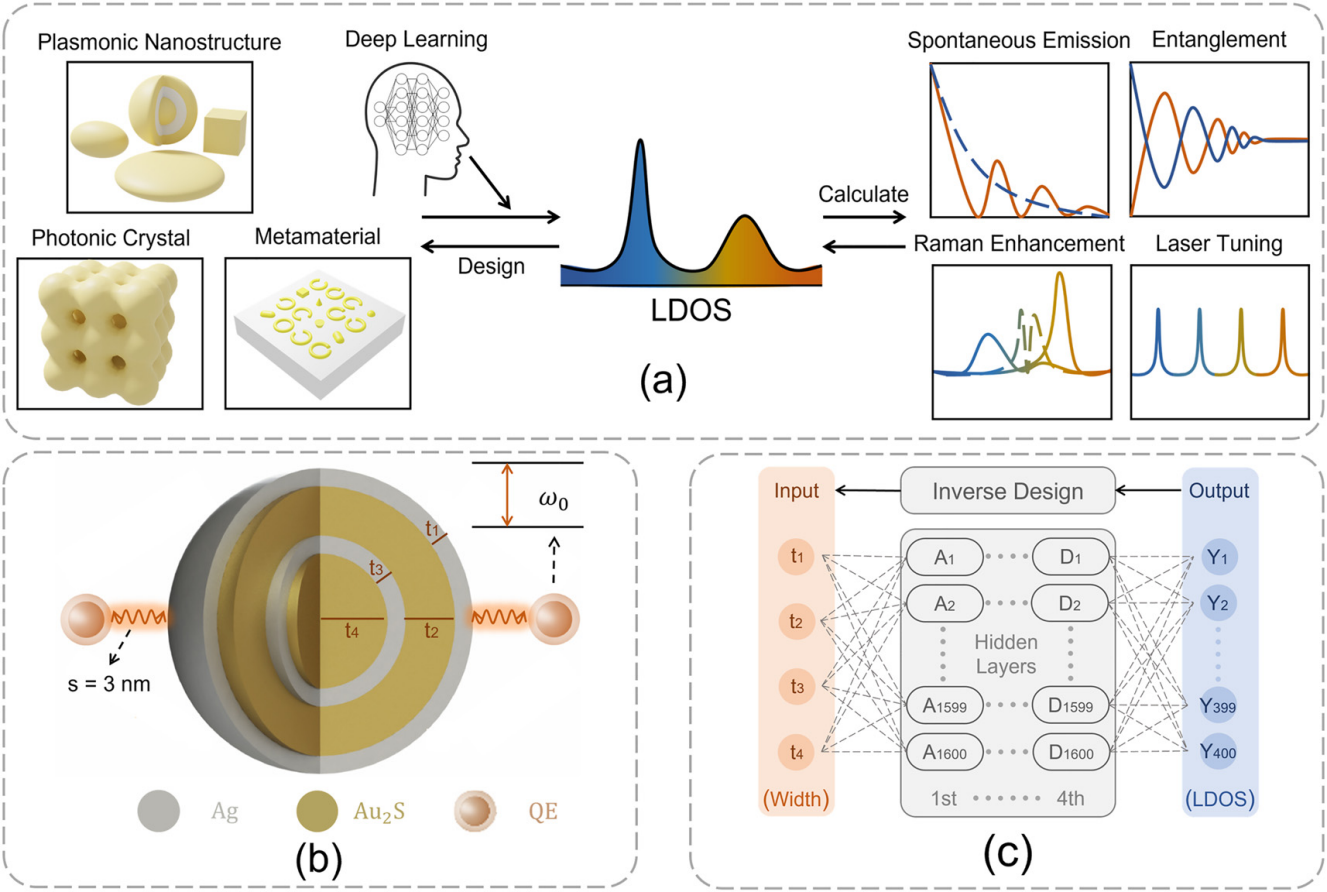
Inverse design in quantum nanophotonics by combining local-density-of-states (LDOS) and deep learning. (a) Generalized framework of inverse design procedure in quantum nanophotonics, where LDOS bridges various quantum characteristics and diversified artificial nanophotonic structures with a deep learning model. (b) A representative quantum nanophotonic system consisting of a multilayer shell–metal–nanoparticle (SMNP) and a pair of quantum emitters (QEs). (c) The fully-connected neural network (NN) with the geometric parameters of a nanophotonic structure (e.g., the layer thicknesses of an SMNP) as input and the LDOS as output.

This work presents a generalized inverse design procedure in quantum nanophotonics by combining LDOS and DL, as illustrated in Figure 1(a). Firstly, artificial nanophotonic structures (such as plasmonic nanostructures [35], photonic crystals [36], and metamaterials [37]) determine the EM environment and its corresponding LDOS, which should be learned by a DL model. Meanwhile, the relationship between the LDOS and the quantum characteristics (e.g., spontaneous emission and entanglement dynamics) [26, 28] or quantum applications (e.g., Raman enhancement and laser tuning) [38, 39] needs to be established. Finally, the “objective” LDOS representing the prospective quantum characteristics and applications is employed to inversely design the desired nanophotonic structures with the trained DL model. This procedure is widely applicable to different quantum nanophotonic inverse-design problems, which are enabled by the LDOS with arbitrary nanophotonic structures. In this work, we exemplify this procedure by considering a representative quantum nanophotonic system (Figure 1(b)), in which QEs (each modeled as a two-level system) are located close to the surface of a multilayer (2-, 4-, 6-, or 8-layer) shell–metal–nanoparticle (SMNP). We inversely design the layer thicknesses of the SMNPs for two quantum nanophotonic problems, i.e., the spontaneous emission from a single QE and the entanglement dynamics of two QEs by adopting a fully-connected NN (Figure 1(c)) to approximate the DL function from the layer thicknesses to LDOS. Our results are promising, extending the nanophotonic inverse-design engineering to the quantum regime for advancing quantum device designs.

## 2 Linking LDOS to quantum characteristics

To optimize the quantum characteristics via LDOS in a quantum nanophotonic system, the first necessary step is to analyze the relationship between the quantum characteristics and LDOS. In our study, we consider one or two QEs (modeled as identical two-level systems) interacting in an EM environment, and trace the dynamic changes in the excited-state population of the QEs, denoted as |*C*_*m*_(*t*)|, i.e., the probability amplitude as a function of time. To establish its relationship with LDOS, we perform Laplace transform (forward and backward Fourier transforms) and obtain its frequency-domain spectral expression $C_{m}^{+}\left(\omega^{+}\right)=\int_{0}^{+\infty }C_{m}\left(t\right){\text{e}}^{\text{i}\omega^{+}t}dt$ and $C_{m}^{-}\left(\omega^{-}\right)=\int_{-\infty }^{0}C_{m}\left(t\right){\text{e}}^{\text{i}\omega^{-}t}dt$ [40–42], where the complex frequencies *ω* ^±^ = *ω* ± i*η* are assumed to contain a real frequency *ω* and an infinitely small imaginary part *η* → 0.

The first quantum characteristic we consider in this study is the spontaneous emission, in which a QE transits from an excited energy state to a lower energy state (e.g., the ground state) and emits a quantized amount of energy in the form of a photon. When the QE is placed close to a multilayer SMNP (*s* = 3 nm as shown in Figure 1(b)), the spontaneous emission from the QE arises due to interaction between the QE and the EM environment created by SMNP. Accordingly, we can derive the spectral expression of the population of the QEs to describe the spontaneous emission dynamics (see Supporting Information S1), which depends on the local coupling strength to the EM environment Γ(*ω*) [41]:

(1) $$C_{\text{e}}\left(\omega \right)=\frac{1}{\pi }\frac{C_{\text{e}0}\cdot \left[\Gamma \left(\omega \right)/2\right]}{{\left(\omega -\omega_{\text{e}}\right)}^{2}+{\left[\Gamma \left(\omega \right)/2\right]}^{2}},$$

where *C*_e0_ = 1 is the initial state of the QE in the excited state, *ω*_e_ is the transition frequency between the two states, and $\Gamma \left(\omega \right)=\left(2\omega^{2}\right)/\left(\hbar \varepsilon_{0}c^{2}\right)\times d\cdot Im\left[G\left(r,r,\omega \right)\right]\cdot d$ . It should be emphasized that *ω*_e_ has taken into account the level shift induced by the environment $\Delta \left(\omega \right)=\left(-\omega^{2}\right)/\left(\hbar \varepsilon_{0}c^{2}\right)\times d\cdot Re\left[G\left(r,r,\omega \right)\right]\cdot d$ , where Δ(*ω*) and Γ(*ω*) satisfy the Kramers–Kronig relation. The term **G**(**r**, **r**, *ω*) is the frequency-dependent EM Green’s function related to the nanophotonic structure, **r** is the location of the QE, and **d** is the transition dipole moment of the QE. We can express Γ(*ω*) as a function of LDOS Γ(*ω*) = (*πωd* ^2^)/(ℏ*ɛ*_0_)*ρ*(**r**, *ω*) with the LDOS written as:

(2) $$\rho \left(r,\omega \right)=\frac{2\omega }{\pi c^{2}}\times n\cdot Im\left[G\left(r,r,\omega \right)\right]\cdot n,$$

where *d* is the magnitude of the transition dipole moment of the QE, and **n** is the unit vector pointing along the direction of the transition dipole moment [43].

For the second quantum characteristic, we consider a pair of QEs labeled as QE_*a*_ and QE_*b*_ forming a quantum entanglement, which occurs when a group of particles (in this case, two QEs) is generated, interact, or share spatial proximity in a way such that the quantum state of each particle of the group cannot be described independently of the state of the others. By tracing the dynamic changes in the population of the two QEs, their entanglement dynamics display the interaction strength between the QEs under the influence of an EM environment. Likewise, their entanglement dynamics can be computed through the spectral expressions [42] (see Supporting Information S2):

(3) $$\begin{array}{c} C_{a}\left(\omega \right)=2Re\left\{\frac{\begin{array}{c} i\left[\left(\omega -\omega_{b}+\frac{i\Gamma_{bb}\left(\omega \right)}{2}\right)C_{a0}\right.\\ \left.+\left(\Delta_{ab}\left(\omega \right)-\frac{i\Gamma_{ab}\left(\omega \right)}{2}\right)C_{b0}\right] \end{array}}{\begin{array}{c} \left(\omega -\omega_{a}+\frac{i\Gamma_{aa}\left(\omega \right)}{2}\right)\left(\omega -\omega_{b}+\frac{i\Gamma_{bb}\left(\omega \right)}{2}\right)\\ -{\left(\Delta_{ab}\left(\omega \right)-\frac{i\Gamma_{ab}\left(\omega \right)}{2}\right)}^{2} \end{array}}\right\},\\ C_{b}\left(\omega \right)=2Re\left\{\frac{\begin{array}{c} i\left[\left(\omega -\omega_{a}+\frac{i\Gamma_{aa}\left(\omega \right)}{2}\right)C_{b0}\right.\\ \left.+\left(\Delta_{ab}\left(\omega \right)-\frac{i\Gamma_{ab}\left(\omega \right)}{2}\right)C_{a0}\right] \end{array}}{\begin{array}{c} \left(\omega -\omega_{a}+\frac{i\Gamma_{aa}\left(\omega \right)}{2}\right)\left(\omega -\omega_{b}+\frac{i\Gamma_{bb}\left(\omega \right)}{2}\right)\\ -{\left(\Delta_{ab}\left(\omega \right)-\frac{i\Gamma_{ab}\left(\omega \right)}{2}\right)}^{2} \end{array}}\right\}. \end{array}$$

Here, we assume *C*_*a*0_ = 1 and *C*_*b*0_ = 0, indicating the initial state of QE_*a*_ in the excited state and QE_*b*_ in the ground state; *ω*_*a*_ and *ω*_*b*_ are the transition frequencies that have taken the level shift of the EM environment into consideration: $\Delta_{mn}\left(\omega \right)=\left(-\omega^{2}\right)/\left(\hbar \varepsilon_{0}c^{2}\right)\times d_{m}\cdot Re\left[G\left(r_{m},r_{n},\omega \right)\right]\cdot d_{n}$ , where *m*, *n* = *a* or *b*. The coupling strength of the EM environment with one QE at the location **r**_*m*_ and the other QE at the location **r**_*n*_ is represented by $\Gamma_{mn}\left(\omega \right)=\left(2\omega^{2}\right)/\left(\hbar \varepsilon_{0}c^{2}\right)\times d_{m}\cdot Im\left[G\left(r_{m},r_{n},\omega \right)\right]\cdot d_{n}$ , in which **G**(**r**_*m*_, **r**_*n*_, *ω*) are the frequency-dependent EM Green’s functions reflecting the local field effect induced by the nanophotonic structure. Since the coupling strength Γ_*mn*_(*ω*) represents the EM field response either at a single point in space or between a pair of points. Another scalar quantity is introduced to represent the spatial correlation between a pair of points in space, i.e., cross-density-of-states (CDOS) [35]. We can express Γ_*mn*_(*ω*) as a function of CDOS: Γ_*mn*_(*ω*) = (*πωd* ^2^)/(ℏ*ɛ*_0_)*ρ*_*mn*_(**r**_*m*_, **r**_*n*_, *ω*) with the CDOS written as:

(4) $$\rho_{mn}\left(r_{m},r_{n},\omega \right)=\frac{2\omega }{\pi c^{2}}\times n_{m}\cdot Im\left[G\left(r_{m},r_{n},\omega \right)\right]\cdot n_{n}.$$

When the QE_*a*_ and QE_*b*_ are symmetrically placed around the multilayer SMNP as shown in Figure 1(b), which means **r**_*a*_ = −**r**_*b*_, all Γ_*mn*_(*ω*) have the same distributions. For *m* = *n*, i.e., Γ_*aa*_(*ω*) and Γ_*bb*_(*ω*) characterize the local coupling strength between a QE and the EM environment created by SMNP, and the CDOS coincide with the LDOS [43].

From the rigorous theoretical analysis above, we prove that the spontaneous emission and entanglement dynamics can be determined exactly by LDOS. We can then manipulate them by inversely designing the nanophotonic structure via LDOS.

## 3 Inverse design nanophotonics from LDOS

After establishing the relationship between the quantum characteristics and LDOS, we will develop the inverse-design procedure to link the “output” LDOS and the “input” nanophotonic structure; in this study, a multilayer (2-, 4-, 6-, or 8-layer) SMNP where the two QEs (QE_*a*_ and QE_*b*_) sit at *s* = 3 nm from the surface of the nanoparticle. Here, the choice of SMNP over the more common metal-nanoparticle (MNP) [25], [26], [27, 44, 45] is due to the flexibility to design the surface plasmon resonances (SPRs) [28, 46], [47], [48] by controlling the number of layers in SMNP and their thicknesses [28, 49, 50], apart from providing humongous field amplifications [51]. In recent years, multilayer SMNPs have been developed for various applications, such as quantum dot [52], nano-laser [53], and fluorescence enhancement [54]. In particular, a forward-design transfer-matrix method has been applied to reveal the critical role of the shell for 2-layer SMNPs in fluorescence enhancement, where the general design guidelines have been provided by scanning over up to 10^5^ different configurations for each wavelength [54]. Thus, it is instructive to develop an effective inverse-design approach to manipulate the optical properties of the multilayer SMNP.

As depicted in Figure 1(b), the multilayer SMNP consists of a metallic Ag shell and alternating dielectric Au_2_S and metallic Ag layers (with their thicknesses labeled as *t*_1_, *t*_2_, *t*_3_, *t*_4_, …). Such a material combination [28] can be readily synthesized [55–57]; and benefits from (i) strong local field enhancement at the metallic Ag surface due to surface plasmon resonance effect [58, 59], and (ii) suitable values of band gap varying from 1.3 ∼ 2.6 eV for the dielectric Au_2_S [60] ensuring the tunability of plasmon resonance of the SMNP. The surrounding of the multilayer SMNP is assumed to be an aqueous medium of dielectric constant *ɛ*_*s*_ = 1.78 [28]. We develop a deep learning (DL) model to design the thickness of each layer in the multilayer SMNP by imposing different thickness limits for the two types of materials (Ag: *ɛ*_1_(*ω*) [61], 1 ∼ 5 nm, Au_2_S: *ɛ*_2_ = 5.4 [28], 1 ∼ 30 nm) to manipulate LDOS in the space near the QEs.

As illustrated in Figure 1(c), we adopt a classical fully-connected NN with four hidden layers and 1600 neurons in each segment. We take the thickness of each layer of the SMNP as the DL input and the *r*-component (radial direction) of the LDOS enhancement at the location of QE with respect to that of the surrounding medium as our DL output and optimization objective, which is defined as [28, 62]:

(5) $$F_{r}\left(\omega \right)=\frac{n_{r}\cdot Im\left[G\left(r,r,\omega \right)\right]\cdot n_{r}}{n_{r}\cdot Im\left[G^{0}\left(r,r,\omega \right)\right]\cdot n_{r}}.$$

Here, $Im\left[G^{0}\left(r,r,\omega \right)\right]=\left(\omega \sqrt{\varepsilon_{s}}\right)/\left(6\pi c\right)$ is the imaginary part of the Green’s tensor in surrounding medium far from the SMNP, and **r** is the location of QE. The enhancement of LDOS can be calculated by solving Maxwell’s equations, in which the analytical solution of multilayer SMNPs is readily available [63, 64] (see Supporting Information S3 for a detailed derivation of the dyadic Green’s function) and taken to generate 5 × 10^5^ samples per dataset with each dataset splitting into 0.8 for training and 0.2 for verification. With this procedure, we have established high-performance DL models for all kinds of multilayer SMNPs: the mean relative accuracy (MRA) >85 % with the trained dataset and >80 % with the verified dataset. With the verified dataset, our DL models for 8-, 6-, 4-, and 2-layer SMNPs achieved the accuracy of 84.02 %, 86.46 %, 91.73 %, and 99.32 % (see Supporting Information S4).

With the DL model ready, we employ the neural-adjoint method [65], which has been used to optimize nanoparticles [9] and metasurfaces [66]. This method, as a gradient-based optimization method, has the problem of getting stuck in the local minima. This can be solved by setting the max and min values of the loss function to select a suitable initial value since different initial values strongly affect the final optimization result. Based on this property, the neural-adjoint method has the advantage of generating multiple optimization results for one design objective with a suitable design loss function. To obtain LDOS enhancement of specific amplitude and width, our design loss function is devised as follows:

(6) $${Loss}_{d}=\overset{m}{\underset{j=1}{\sum }}\frac{w^{j}}{n}means\overset{n}{\underset{i=1}{\sum }}\left(\frac{{E_{x}}_{i}^{j}}{sum\left({E_{x}}_{i}^{j}\right)}\left|Y_{i}-{E_{x}}_{i}^{j}\right|\right),$$

where *n* is the number of output points, *m* is the number of objectives, *Y* is the DL output (e.g., the amplitude of LDOS enhancement), *E*_*x*_ is the objective (e.g., the predefined amplitude of LDOS enhancement), and *w* is the weightage of the objectives. This design loss function offers improved efficiency than those conventional loss functions such as mean-squared-error (MSE) and mean-absolute-error (MAE) for studies with large output variation [67, 68] because the value of output points determines their weights, which means that some output points with relatively small values will be neglected to accelerate calculation. We can also set multiple objectives in the same designing process, which can be combined with the weightage parameter *w*. More details about the inverse-design algorithm can be found in the Supporting Information S4.

**Figure 2:**
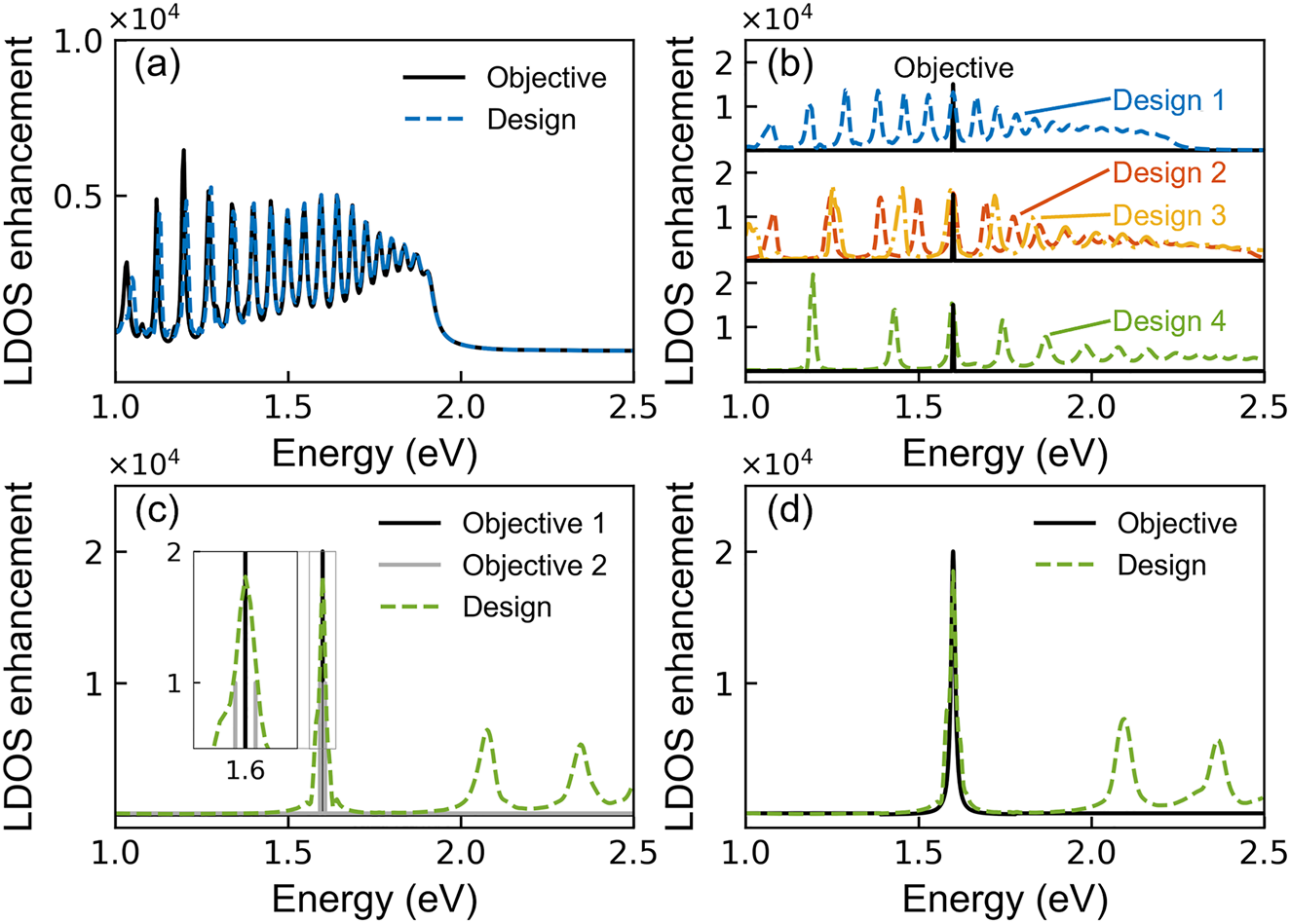
Inverse designed multilayer SMNP to meet different objectives. (a) An “expected” objective that resembles a typical forward-calculation results from our dataset and an optimized 8-layer SMNP design. (b) An “ideal” point-like objective representing the desired feature of LDOS with absolutely sharp single-resonant-frequency and multiple optimized “designs” of 8-layer SMNP (design 1), 6-layer SMNP (designs 2 and 3), and 4-layer SMNP (design 4). (c) and (d) Two methods to optimize spectral shape (or the *Q*-factor) of a resonant mode for a 4-layer SMNP by using (c) “multi-points” to represent multiple objectives or (d) Lorentz function.

Based on this back-propagation algorithm, we inversely design our SMNPs with a different number of layers (e.g., 8-, 6-, or 4-layer) to meet different objectives and demonstrate a few representative examples in Figure 2. These inversely-designed SMNPs will be used later for our quantum nanophotonic applications. First, we consider two different types of objectives and compare them in Figure 2(a) and (b) (the black solid lines). Figure 2(a) illustrates a case study with an “expected” objective, which resembles a typical forward-calculation result by the analytical solution displaying multiple resonant frequencies but is not within our dataset. In contrast, Figure 2(b) demonstrates a case study with an “ideal” point-like objective representing the desired feature of LDOS with a sharp single resonant frequency. In many quantum nanophotonic applications, such a single resonant frequency of the nanophotonic structure is desirable to match the transition frequency of the QEs to achieve zero detuning [69–71]. At the resonant condition, one can manipulate the amplitude of LDOS enhancement at the resonant frequency to realize strong coupling that significantly changes the quantum characteristics [72].

With these two distinct objective functions, we inversely design the SMNPs and present our best-optimized “designs” on top of the “objectives”. As expected, the optimized “design” (dashed-line), an 8-layer SMNP, has an LDOS enhancement spectrum almost perfectly aligning with the “expected” objective (solid-line) as evidenced in Figure 2(a). However, it is more challenging to find an optimized “design” with the LDOS enhancement matching exactly with the “ideal” point-like objective over the whole spectrum from 1 to 2.5 eV in Figure 2(b). Instead, multiple optimized “designs” of 8-layer SMNP (design 1), 6-layer SMNP (designs 2 and 3), and 4-layer SMNP (design 4) can simultaneously meet the requirement of the “objective” at the single objective resonant frequency with the desired amplitude of LDOS enhancement, though all these “designs” also provide extra but unwanted resonant peaks at the side.

During our designing process, we found that the increased number of layers for SMNP will generate more resonant peaks closely packed together, as displayed in Figure 2(b). Such closely packed resonant peaks will reduce the efficiency of energy confinement at the desired resonant mode, causing the energy to leak out to the neighboring modes easily (equivalent to bandwidth broadening). This, in turn, decreases the quality factor (*Q*-factor) of the desired resonant mode, defined as the ratio of a resonator’s center frequency to its bandwidth or full width at half maximum (FWHM), which may induce a crucial impact in many quantum applications, e.g., the lifetime or stability of the quantum characteristics [73].

To take the resonance shaping (or *Q*-factor engineering) into our inverse-design process, it is instructive to predefine the spectral width of the LDOS enhancement as the objective function in addition to the amplitude. We propose two methods, both of which add small values as non-zero weights over the whole spectrum to reduce extra resonant peaks that may decrease the *Q*-factor. The first method uses “multi-points” to represent multiple objectives as plotted in Figure 2(c) (solid-lines: black and grey). To be more specific, we set a central point at 1.6 eV with a value reaching 2 × 10^4^ (black solid-line) to define the amplitude of the LDOS enhancement, and the other two points reaching 1 × 10^4^ located at 1.6075 eV and 1.5925 eV (grey solid-lines) to define the spectral width of the LDOS enhancement. We successfully designed a 4-layer SMNP (dashed-line) with its spectral width and frequency amplitude simultaneously meeting the two objectives within the same step with divided loss errors and different limitations. A zoom-in comparison can be seen clearly in the Inset. The second method (Figure 2(d)) takes a Lorentz function $L\left(\omega \right)=\frac{1}{2}\Delta \omega /\left[{\left(\omega -\omega_{r}\right)}^{2}+{\left(\frac{1}{2}\Delta \omega \right)}^{2}\right]$ , where *ω*_*r*_ is the objective resonant frequency and Δ*ω* is the FWHM [74]. A Lorentz function is defined as our objective (black solid line), in which the peak at *ω*_*r*_ = 1.6 eV reaches 2 × 10^4^, and the FWHM is Δ*ω* = 0.015 eV, which is comparable to the three-point objective in Figure 2(c). As clearly shown in Figure 2(d), an optimized 4-layer SMNP design (dashed-line) can reasonably well resemble the objective Lorentz function around 1.6 eV, while the other resonant modes (above 2.0 eV) are also moderately far away from the objective resonant frequency. In short, the two proposed methods both effectively optimize spectral width (or *Q*-factor), amplitude, and resonant frequency. Since the first multiple-point method does not consider a regular shape of the output spectrum, it can be more widely applicable to other problems with asymmetrical resonance shapes. Using multiple point-like objectives to shape the resonance is generally more flexible.

## 4 Quantum nanophotonic applications

Up to this point, we have linked the “bridge” LDOS with the quantum characteristics and the inverse-design nanophotonics. We are now ready to apply the inverse-design procedure to design our nanophotonic structure, the SMNP, to optimize the quantum characteristics in two quantum nanophotonic applications mentioned above: (i) the spontaneous emission from a single QE and (ii) the quantum entanglement between two QEs, where QEs are sitting close to the SMNP in all studies.

### 4.1 Optimizing spontaneous emission

Spontaneous emission plays a significant role in determining the performance of many quantum nanophotonic devices [75, 76]. Spontaneous emission behaves differently in different coupling regimes characterizing the interaction between the QEs and the nanophotonic structure: weak- and strong-coupling [77]. In the weak-coupling regime, only the spontaneous emission rate is modified, while the emission frequency equalling the transition frequency of the QE remains unaltered. However, if the interaction is strong enough, the emission frequency is also changed and becomes inextricably linked with the EM environment created by the nanophotonic structure, known as the strong-coupling regime. According to our theoretical analysis in Eq. (1), the local coupling strength Γ(*ω*) characterizes the interaction of the QE with the photonic modes at any frequency, valid for both weak- and strong-coupling regimes [41]. By considering an ideal single resonant frequency without loss, the local coupling strength can be simplified to Γ(*ω*) = 2*π*|*g*_*r*_|^2^ *δ*(*ω* − *ω*_*r*_), where *ω*_*r*_ is the resonant frequency and *g*_*r*_ is the coupling coefficient that satisfies $g_{r}=-i\sqrt{\left(\omega_{r}\right)/\left(2\hbar \varepsilon_{0}\right)}\times d\cdot E\left(r,\omega_{r}\right)$ with the electric field **E**(**r**, *ω*_*r*_) at the resonant frequency *ω*_*r*_ [41, 78]. Here, the coupling coefficient *g*_*r*_ exactly equals the Rabi frequency in the form of **d** ⋅ **E**/ℏ, with a normalization constant $-i\sqrt{\left(\hbar \omega_{r}\right)/\left(2\varepsilon_{0}\right)}$ [79]. Substituting this Γ(*ω*) into Eq. (1), we reveal two factors to achieve strong coupling: (i) the matching between the resonance of the quantum nanophotonic structure with that of the QE (*ω*_*r*_ = *ω*_*e*_); and (ii) the high value of the local coupling strength Γ(*ω*_*r*_) at the resonant frequency *ω*_*r*_, which is proportional to the amplitude of the LDOS *ρ*(**r**, *ω*) for realistic cavities with energy leakage. Therefore, the spontaneous emission can be manipulated by designing the resonant frequency and amplitude of the LDOS enhancement.

**Figure 3:**
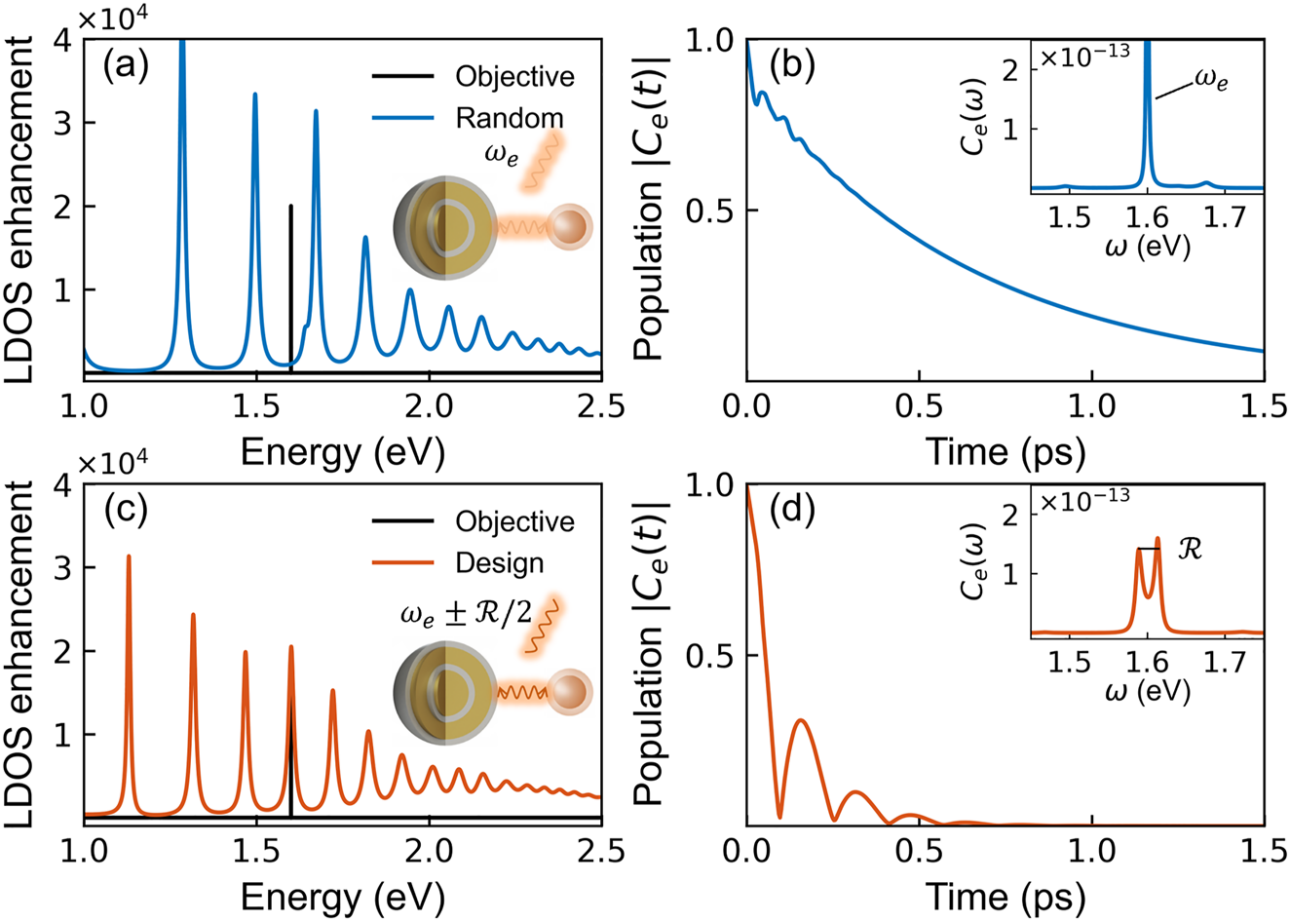
Optimizing spontaneous emission dynamics in a quantum nanophotonic system consisting of a 4-layer SMNP and a single QE. Comparison between unoptimized random structure (*t*_1_ = 1.02 nm, *t*_2_ = 11.20 nm, *t*_3_ = 1.52 nm, and *t*_4_ = 4.40 nm) (upper panel) and optimized design structure (*t*_1_ = 1.21 nm, *t*_2_ = 20.84 nm, *t*_3_ = 2.20 nm, and *t*_4_ = 4.18 nm) (lower panel) to achieve strong coupling between the SMNP and the QE. (a) and (c) Plot the LDOS enhancement for the corresponding SMNP; (b) and (d) plot the dynamic changes in the excited-state population of the QE |*C*_e_(*t*)| and its frequency-domain spectrum *C*_e_(*ω*) (Inset).

We consider a single QE (transition frequency *ω*_*e*_ = 1.6 eV, negligible decay rate, the magnitude of transition dipole moment *d* = 60 D), optimize its spontaneous emission dynamics when the QE is placed at *s* = 3 nm away from a 4-layer SMNP by designing the LDOS of the SMNP. We employ a point-like objective function, a central point at 1.6 eV with a value reaching 2 × 10^4^ (black solid-line) to represent QE, as shown in Figure 3. The upper panel (Figure 3(a) and (b)) shows the results of a random SMNP structure without optimization (*t*_1_ = 1.02 nm, *t*_2_ = 11.20 nm, *t*_3_ = 1.52 nm, and *t*_4_ = 4.40 nm), whereas the lower panel (Figure 3(c) and (d)) demonstrates the results of an optimized SMNP design (*t*_1_ = 1.21 nm, *t*_2_ = 20.84 nm, *t*_3_ = 2.20 nm, and *t*_4_ = 4.18 nm).

Comparing the LDOS enhancement of the SMNP, the random SMNP structure (Figure 3(a)) exhibits an apparent detuning between the QE (represented by the objective function) and the SMNP, while the optimized design (Figure 3(c)) can provide a resonant mode matching with the QE. Intuitively, the coupling between the random SMNP and QE could be weak, and the emission frequency remains as the transition frequency of QE (see Inset of Figure 3(a)). In contrast, the optimized SMNP design can be strongly coupled to QE, whose emission frequency splits by the amount of vacuum Rabi frequency $R=2g_{r}$ [80, 81], indicating the coupling strength between QE and SMNP (see Inset of Figure 3(c)).

To prove it, we quantitatively characterize the spontaneous emission dynamics by calculating the population |*C*_e_(*t*)| of the QE as a function of time and its spectrum *C*_e_(*ω*) (Insets), as shown in Figure 3(b) and (d). The population dynamics of the QE sitting around the random SMNP (Figure 3(b)) show relatively smooth attenuation with slight oscillations initially, and the corresponding spectrum *C*_e_(*ω*) shows only a single emission peak at *ω*_e_, clear evidence of weak-coupling. On the other hand, the population of the same QE sitting around the optimized SMNP (Figure 3(d)) displays several apparent oscillations, and the corresponding spectrum *C*_e_(*ω*) shows two clear peaks with almost the same height, i.e., Rabi-splitting, signifying the strong coupling regime [82–84]. Such fast oscillation may be beneficial to developing a single photon source based on spontaneous emission in terms of brightness, corresponding to the high repetition rate for the generation of single photons in each pulse [85]. In short, regulating the coupling strength between a QE and an SMNP and the corresponding spontaneous emission dynamics is feasible by manipulating the LDOS (i.e., the amplitude at the objective frequency) via inversely designing the SMNP.

### 4.2 Optimizing entanglement dynamics

Quantum entanglement describes a group of particles interacting under a single quantum state. As a significant quantum characteristic, it can be formed between two QEs under a local EM environment caused by the nanophotonic structure [86, 87]. In this example, we consider two identical QEs (transition frequency *ω*_*a*_ = *ω*_*b*_ = 1.6 eV, negligible decay, the magnitude of transition dipole moment *d* = 60 D) sitting at the opposite side of the SMNP and *s* = 3 nm away from the metal surface. We can optimize the quantum entanglement between the two QEs by engineering the local EM environment described as LDOS. Due to the symmetry of the QEs’ locations, the LDOS at the two QEs is the same, and only the LDOS of one QE needs to be optimized. Commonly, a dimensionless parameter R=R/Δω is used to characterize the nanophotonic structures for efficient quantum entanglement, where $R=2g_{r}$ is the amount of vacuum Rabi frequency [80, 81] and Δ*ω* is the FWHM [88]. An ideal nanophotonic structure (*R* ≫ 1, at least *R* = 10) [88] requires high amplitude of the LDOS enhancement for higher coupling strength *g*_*r*_ and small FWHM at resonance. As FWHM is essentially the same as the Q-factor *Q* = *ω*_*r*_/Δ*ω* [89], manipulating the quantum entanglement with the LDOS should consider the two factors: high amplitude and high *Q*-factor.

First of all, we reveal the effect of the amplitude of the LDOS enhancement on the entanglement dynamics by comparing the two SMNPs in Figure 3 (see Supporting Information S5). The results indicate that a higher amplitude of the LDOS enhancement at the objective frequency (the optimized SMNP design in Figure 3(c)) leads to a smaller oscillation period and faster entanglement, indicating a stronger interaction. This fact suggests that the SMNP can enhance the interaction between the QEs. Meanwhile, the SMNP also introduces a decay channel crucial for quantum entanglement. The spectral width of the designed resonant mode and those closely packed resonant peaks are responsible for the decay, which the *Q*-factor characterizes. Therefore, the decay rate of the entanglement dynamics could be optimized by *Q*-factor engineering.

**Figure 4:**
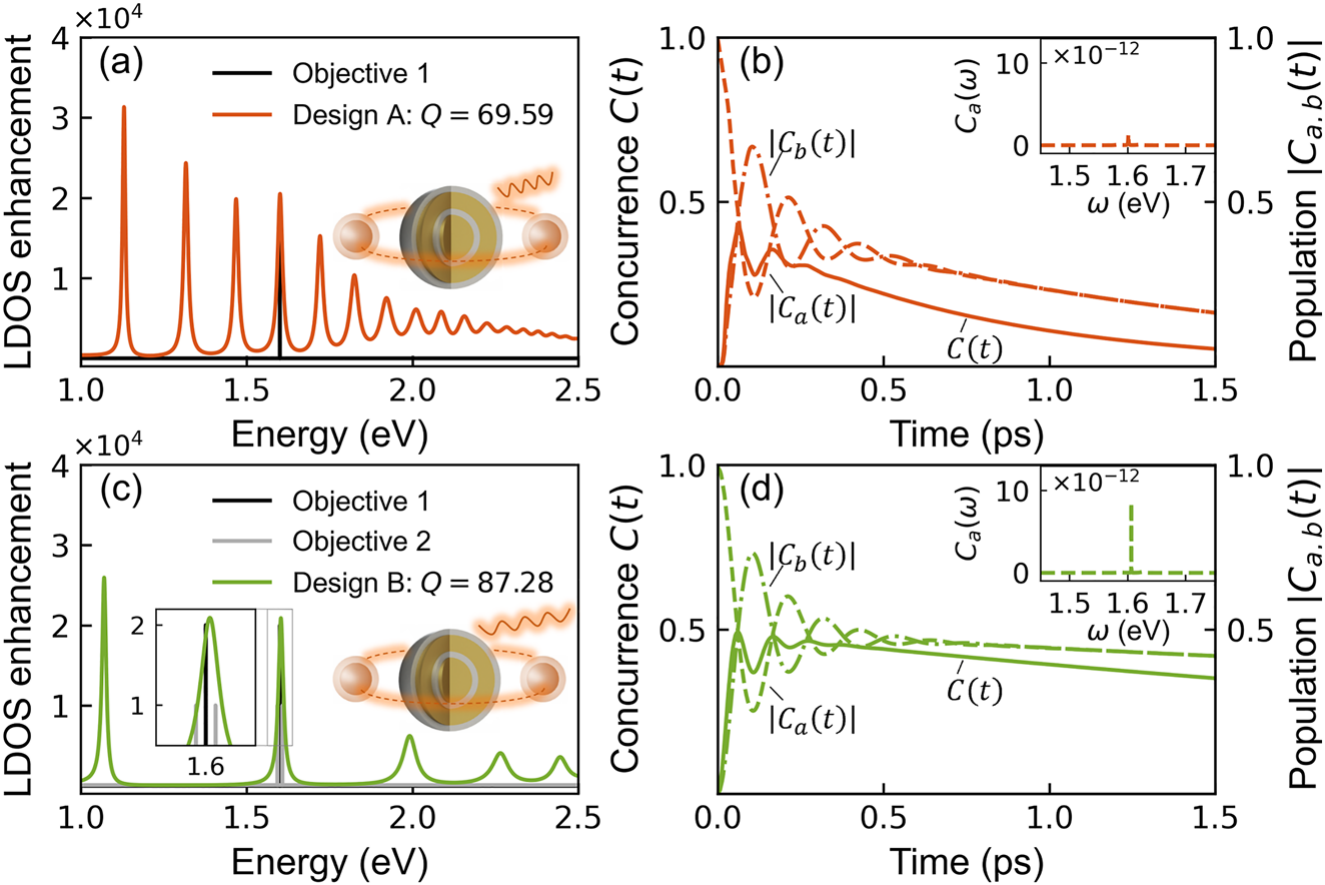
Optimizing decay rate in entanglement dynamics for a quantum nanophotonic system consisting of a 4-layer SMNP and two QEs (QE_*a*_ and QE_*b*_). Comparison of the two optimized design structures (A and B) by optimizing only the resonant frequency and amplitude (upper panel) (*t*_1_ = 1.21 nm, *t*_2_ = 20.84 nm, *t*_3_ = 2.20 nm, and *t*_4_ = 4.18 nm with the *Q*-factor *Q* = 69.59) or by optimizing the resonant frequency, amplitude, and spectral width (lower panel) (*t*_1_ = 1.84 nm, *t*_2_ = 3.73 nm, *t*_3_ = 4.65 nm, and *t*_4_ = 3.45 nm with the *Q*-factor *Q* = 87.28). (a) and (c) Plot the LDOS enhancement for the corresponding SMNP. (b) and (d) Plot the concurrence *C*(*t*) (solid line) characterizing entanglement dynamics; the dynamic changes in the excited-state population of the two QEs for |*C*_*a*_(*t*)| (dashed lines) and |*C*_*b*_(*t*)| (dashed-dotted lines); and the spectrum of *C*_*a*_(*ω*) for QE_*a*_ (Inset).

We take the “point-like” objective approach to optimize the decay rate of the entanglement dynamics, and the results are compared in Figure 4. The upper panel (Figure 4(a) and (b)) shows the results optimizing the resonant frequency and amplitude of the LDOS enhancement with a single-point objective (black line), whereas the lower panel (Figure 4(c) and (d)) shows the results not only optimizing the resonant frequency and amplitude but also the spectral width of the LDOS enhancement, by using three-point objectives (black and grey lines). Clearly, with the single-point or three-point objectives imposed, both designs A (Figure 4(a)) and B (Figure 4(c)) can generate a resonant mode at the desired frequency of 1.6 eV with similar amplitude 2 × 10^4^. However, the *Q*-factors of the two resonances differ. Design A with a single-point objective achieved *Q* = 69.59, while design B with a three-point objective reached *Q* = 87.28, implying a potentially more stable entanglement (Insets of Figure 4(a) and (c)).

To quantify the quantum entanglement between the two QEs, the concurrence is often used and defined as $C\left(t\right)=2\max\left[0,|C_{a}\left(t\right)C_{b}^{*}\left(t\right)|\right]$ , ranging from zero for separable states up to 1 for maximally entangled states, where the slope of *C*(*t*) represents the decay rate of the entanglement [28]. Together with the population dynamics |*C*_*a*,*b*_(*t*)| of the two QEs, we plot the concurrence dynamics *C*(*t*) in Figure 4(b) and (d), where the corresponding spectrum *C*_*a*_(*ω*) for one of QEs are drawn in the Insets. Firstly, the concurrence and the population for design A with a smaller *Q*-factor (Figure 4(b)) generate a faster attenuation, *i*.*e*., the slope is shaper; while the curves for design B with a larger *Q*-factor (Figure 4(d)) generally look flatter, and they saturate close to a relatively large value of 0.5. Besides, the spectra *C*_*a*_(*ω*) (Inset) display a much higher amplitude in the high-*Q* result (Figure 4(d)) than the low-*Q* result (Figure 4(b)), representing lower decay rate of the entanglement in the frequency domain. These features prove that the entanglement’s decay rate can be optimized by manipulating the *Q*-factor of the LDOS enhancement. Similar conclusions can be drawn based on the Lorentz function approach; meanwhile, different types of SMNPs (e.g., 2-layer) can be similarly optimized with these design methods (see Supporting Information S6).

### 4.3 Experimental considerations

It is still an experimental challenge to fabricate multilayer SMNP with an accurate design of layer thickness, which is usually produced via layer-by-layer growth such as chemical vapor deposition (CVD) [90, 91]. To address this problem, we propose smart manufacturing as a potential solution, which involves computer-integrated manufacturing, high levels of adaptability, and rapid design changes. First of all, we provide a reference structure for fabrication. As the inner layer of SMNP is grown, we can measure its thickness and inverse design a new structure accordingly. After some adaptive cycles, a different structure with the same optical properties as the reference structure can be produced. Our proposed solution requires re-calculations with the determined layer thickness, essentially obtaining multiple optimal structures with “partially fixed thicknesses”, which can be addressed by the inverse design framework. For example, based on the designed structures in Figure 4(c) (*t*_1_, *t*_2_, *t*_3_, *t*_4_) = (1.84, 3.73, 4.65, 3.45), we find two other structures (1.83, 13.52, 1.72, 3.45) and (1.93, 10.91, 4.65, 3.45) with partially fixed thicknesses of the most inner layer *t*_4_ = 3.45 nm; or the two inner layers *t*_3_ = 4.65 nm and *t*_4_ = 3.45 nm. All three designs have consistent LDOS enhancement and entanglement dynamics (see Supporting Information S7). In short, our inverse-design framework is a flexible computer design tool that can be integrated into manufacturing.

## 5 Conclusions and outlook

In conclusion, we demonstrated an efficient inverse-design procedure to solve and optimize quantum nanophotonic problems by combining the concept of LDOS and deep learning (DL) techniques. We utilized LDOS, the number of EM field modes per unit volume and frequency at a given point in space, as the optimizing objectives to connect the complex quantum characteristics and the geometries of the nanophotonic structures; and leveraged advanced DL models to establish the function from the geometric parameters to LDOS for nanophotonic structures. We exemplify the procedure on a simple quantum nanophotonic system consisting of QEs sitting on a single multilayer SMNP by adopting a fully-connected NN to model the function from the layer thicknesses to the LDOS. By devising a design loss function and setting simple but robust “multi-point” objectives, we successfully design suitable SMNP to optimize the spontaneous emission from a single QE or quantum entanglement dynamics between two QEs. This work extended nanophotonic inverse-design engineering to the quantum regime.

It is worth highlighting that our inverse-design procedure combined the merits of LDOS and DL. Conventional inverse-design DLs in nanophotonics usually target solving specific problems and structures [92, 93], whereas our LDOS-bridged procedure simplifies complex problems to reduce learning difficulty and can be applied to different problems. Thanks to the development in the DL field, readily available advanced DL models enabled us to approximate more diversified functions and calculate more efficiently than the traditional inverse-design approaches [94–96]. Still, it is more challenging to develop the DL model to approximate and optimize LDOS as compared to the commonly seen classic properties such as scattering spectra [9] or electromagnetic dipole moments [13], because the amplitude of LDOS is not normalized and varies by several orders of magnitude. It means that bigger DL models and larger learning datasets are required. Our proposed design loss function effectively solved the problem as the value of output points determines their weights, which neglected some output points with small values to reduce the computational load. Moving forward, we can extend the current DL model to consider the polarization and chirality of LDOS [97, 98].

Regarding the quantum nanophotonic applications, we have demonstrated the inverse design procedure in spontaneous emission and entanglement dynamics. However, the distances between the QEs and the SMNP are not varied in our present DL model [28, 88], which limits the performance of the concurrence in the two-QE quantum nanophotonic system. Expanding the range of the geometric parameters will be an effective path to enlarge the capacity of the DL model, which in turn, better optimizes the quantum characteristics. Meanwhile, the same LDOS-based procedure can be applied to the quantum nanophotonic systems consisting of more QEs [99, 100], or other near-field applications such as energy transfer [95], photon extractor [96], and surface-enhanced Raman scattering [101, 102], whenever the EM environment created by the nanophotonic structure plays an important role. The essential task would be linking the functional characteristics to LDOS.

Moving forward, our proposed general inverse design framework should be applied to experimental work demonstrating the economy of design with realistic nanoparticles and quantum emitters; alternatively, the unearthing of unconventional or unexpected phenomena. A possible example could be determining how critical the required symmetry in positioning the two quantum emitters is to achieve the computed entanglement. When realizing the system experimentally, the precision in placing the emitters is no easy matter, let alone the nanoparticle shell’s thicknesses.

## Supplementary Material

Supplementary Material Details
