# Supplementary material for: Inverse design in quantum nanophotonics: combining local-density-of-states and deep learning
Source: Nanophotonics. 2023 Apr 13;12(11):1943–55. doi: 10.1515/nanoph-2022-0746 (PMC11501149; doi:10.1515/nanoph-2022-0746)
Supplement: Supplementary file 1 — Supplementary Material Details [file j_nanoph-2022-0746_suppl_001.docx]

Inverse design in quantum nanophotonics based on local density of states: supporting information

Guang-Xin Liu,^†^ Wen-Jie Zhou,^‡^ Ling-Yan Li,^†^ Chun-Lian You,^†^ Jing-Feng Liu,^∗,†^
Cheng-Wei Qiu,^∗,¶^ and Lin Wu^∗,‡,§^
†College of Electronic Engineering and college of artificial intelligence, South China
Agricultural University, Guangzhou 510642, China
‡Science, Mathematics and Technology (SMT), Singapore University of Technology and
Design (SUTD), 8 Somapah Road, Singapore 487372
¶Department of Electrical and Computer Engineering, National University of Singapore, 4
Engineering Drive 3, Singapore 117583
§Institute of High Performance Computing, Agency for Science, Technology, and Research
(A*STAR), Singapore 138632

E-mail: [liujingfeng@scau.edu.cn](mailto:liujingfeng@scau.edu.cn); [chengwei.qiu@nus.edu.sg](mailto:chengwei.qiu@nus.edu.sg); [lin_wu@sutd.edu.sg](mailto:lin_wu@sutd.edu.sg)

1. **Derivation** of **Spontaneous Emission Dynamics**

The basic condition under study considers quantum emitters (QEs) interacting in an EM environment induced by a coupled nanophotonic structure, modelled as quantum nanophotonic systems. The spontaneous emission dynamics as the primary quantum property is studied in the quantum nanophotonic system consisting of a QE and a nanophotonic structure. It is usually represented by the excited-state population $\left| C_{e}(t) \right|$, where $C_{e}(t)$ is the probability amplitude. In our model, we deduce a formalism to calculate the temporal evolution of the probability amplitude $C_{e}(t)$. By adopting the dipole approximation and the rotating-wave approximation, the Hamiltonian of the quantum nanophotonic system can be expressed as^1–3^

$$\begin{aligned} \hat{\mathbf{H}}&=\int d^{3}\boldsymbol{r}\int_{0}^{\infty} \hbar\omega{\hat{\boldsymbol{f}}}^{+}\left( \boldsymbol{r},\omega\right)\hat{\boldsymbol{f}}\left( \boldsymbol{r},\omega\right)d\omega+\hbar\omega_{0}\left| e \right\rangle\left\langle e \right| \\ &+\int_{0}^{\infty} d\omega\left[ \sigma^{+}\mathbf{d}\cdot{\hat{\mathbf{E}}}^{+}\left( \boldsymbol{r},\omega\right)+H.c. \right].\#\left( S1 \right) \end{aligned}$$

Here, the first term represents the field energy of the environment in the presence of the nanophotonic structures. $\hat{\boldsymbol{f}}\left( \boldsymbol{r},\omega\right)$ and ${\hat{\boldsymbol{f}}}^{+}\left( \boldsymbol{r},\omega\right)$ are the bosonic fields that represent the elementary (energy) excitations of the electromagnetic field both in the environment and the nanophotonic structures^4^. The second term in the Hamiltonian represents the energy of the two quantum emitters. $\omega_{0}$and $\mathbf{d=}d\mathbf{n}$ are the transition frequency and transition dipole moment with magnitude $d$ and direction $\mathbf{n}$, respectively, between the excited state $\left| e \right\rangle$ and the ground state $\left| g \right\rangle$ of the quantum emitter located at $\boldsymbol{r}$. The third term in the Hamiltonian represents the interaction between the quantum emitters and the field excitations. $\sigma^{+}$is Pauli operator of the QE. ${\hat{\mathbf{E}}}^{+}\left( \boldsymbol{r},\omega\right)$ is the electric field operator in the frequency domain^4^. It can be expressed as^5^

$$\begin{aligned} \hat{\mathbf{E}}\left( \boldsymbol{r},\omega\right)=i\sqrt{\frac{\hbar}{\pi\epsilon_{0}}}\int d\boldsymbol{r}^{\boldsymbol{'}}\frac{\omega^{2}}{c^{2}}\sqrt{\varepsilon_{I}\left( \boldsymbol{r}^{'},\omega\right)}\mathbf{G}\left( \boldsymbol{r},\boldsymbol{r}^{'},\omega\right)\hat{\boldsymbol{f}}\left( \boldsymbol{r}^{'},\omega\right).\#\left( S2 \right) \end{aligned}$$

Here, $\varepsilon_{I}\left( \boldsymbol{r},\omega\right)$is the imaginary part of the complex relative permittivity $\varepsilon\left( \boldsymbol{r},\omega\right)$ of the metallic nanostructure. $\mathbf{G}\left( \boldsymbol{r},\boldsymbol{r}^{'},\omega\right)$ is the classical Green function (tensor), describing the system response at $\boldsymbol{r}$ to a point source at $\boldsymbol{r}^{\boldsymbol{'}}$, and satisfying the equation $\left[ \frac{\omega^{2}}{c^{2}}\varepsilon\left( \boldsymbol{r},\omega\right)-\nabla\times\nabla\times\right]\mathbf{G}\left( \boldsymbol{r},\boldsymbol{r}^{'},\omega\right)=-\boldsymbol{\delta}\left( \boldsymbol{r}-\boldsymbol{r}^{'} \right)$ together with the boundary condition at infinity. $\boldsymbol{\delta}\left( \boldsymbol{r}-\boldsymbol{r}^{'} \right)$ is the dyadic function. In addition, the time evaluation can be expressed as

$$\begin{aligned} \left| \Psi\left( t \right) \right\rangle=C_{e}\left( t \right)\left| e \right\rangle\left| 0 \right\rangle+\int d^{3}\boldsymbol{r}\int_{0}^{\infty} d\omega C_{g}\left( \boldsymbol{r},\omega,t \right)\left| g \right\rangle\left| \boldsymbol{1}\left( \boldsymbol{r},\omega\right) \right\rangle,\#\left( S3 \right) \end{aligned}$$

where, $\left| 0 \right\rangle$ is the vacuum state of the fields, and $\left| \boldsymbol{1}\left( \boldsymbol{r},\omega\right) \right\rangle$ is the single photon Fock state of the electromagnetic fields. In what follows we assume that the emitter is initially (at time *t* = 0) prepared in the upper state $\left| e \right\rangle$ and electromagnetic field is prepared in the vacuum state $\left| 0 \right\rangle$. So, the initial conditions can be written as $C_{e0}=1$ and $C_{g0}=0$.

By substituting Eqns. (S1), (S2) and (S3) into Schrödinger equation $i\hbar\frac{\partial}{\partial t}\left| \Psi\left( t \right) \right\rangle=\hat{\mathbf{H}}\left| \Psi\left( t \right) \right\rangle$, and considering the orthonormal relationship among $\left| e \right\rangle\left| 0 \right\rangle$, and $\left| g \right\rangle\left| \boldsymbol{l}\left( \boldsymbol{r},\omega\right) \right\rangle$, we can obtain

$$\begin{aligned} \dot{C}_{e}\left( t \right)&=-i\omega_{0}C_{e}\left( t \right) \\ &-\frac{1}{\sqrt{\hbar\pi\epsilon_{0}}}\int_{0}^{\infty} \text{d}\omega\frac{\omega^{2}}{c^{2}}\int\boldsymbol{r}^{\boldsymbol{'}}d\boldsymbol{r}^{\boldsymbol{'}}\sqrt{\epsilon_{I}\left( \boldsymbol{r},\omega\right)}\mathbf{d}\cdot\mathbf{G}\left( \boldsymbol{r},\boldsymbol{r}^{'},\omega\right)C_{g}\left( \boldsymbol{r},\omega,t \right),\#\left( S4a \right) \\ \dot{C_{g}}\left( \boldsymbol{r},\omega,t \right)&=-i\omega C_{g}\left( \boldsymbol{r},\omega,t \right) \\ &+C_{e}\left( t \right)\frac{1}{\sqrt{\hbar\pi\epsilon_{0}}}\frac{\omega^{2}}{c^{2}}\sqrt{\epsilon_{I}\left( \boldsymbol{r}^{\boldsymbol{'}},\omega\right)}\mathbf{d}\cdot\mathbf{G}^{\mathbf{*}}\left( \boldsymbol{r},\boldsymbol{r}^{'},\omega\right).\#\left( S4b \right) \end{aligned}$$

We take the Laplace transform (forward and backward Fourier transform) to transform Eqns. (S4a) - (S4b) into algebraic equations. The forward and backward Fourier transform^6–8^ of any time-dependent variable satisfies the relations as $C_{e}^{+}\left( \varpi\right)=\int_{0}^{+\infty} C_{e}\left( t \right)e^{i\varpi^{+}t}dt$ and $C_{e}^{-}\left( \varpi\right)=\int_{-\infty}^{0} C_{e}\left( t \right)e^{i\varpi^{-}t}dt ADDIN ZOTERO\_ITEM CSL\_CITATION \{"citationID":"4NmjE8oK","properties":\{"formattedCitation":"\backslash\backslash super 6\backslash\backslash uc0\backslash\backslash u8211\{\}8\backslash\backslash nosupersub\{\}","plainCitation":"6-8","noteIndex":0\},"citationItems":[\{"id":247,"uris":["http://zotero.org/users/9173730/items/63SV35YU"],"itemData":\{"id":247,"type":"article-journal","container-title":"Physical Review B","DOI":"10.1103/PhysRevB.87.195138","ISSN":"1098-0121, 1550-235X","issue":"19","journalAbbreviation":"Phys. Rev. B","language":"en","page":"195138","source":"DOI.org (Crossref)","title":"Ab initio determination of local coupling interaction in arbitrary nanostructures: Application to photonic crystal slabs and cavities","title-short":"Ab initio determination of local coupling interaction in arbitrary nanostructures","URL":"https://link.aps.org/doi/10.1103/PhysRevB.87.195138","volume":"87","author":[\{"family":"Chen","given":"Gengyan"\},\{"family":"Yu","given":"Yi-Cong"\},\{"family":"Zhuo","given":"Xiao-Lu"\},\{"family":"Huang","given":"Yong-Gang"\},\{"family":"Jiang","given":"Haoxiang"\},\{"family":"Liu","given":"Jing-Feng"\},\{"family":"Jin","given":"Chong-Jun"\},\{"family":"Wang","given":"Xue-Hua"\}],"accessed":\{"date-parts":[["2022",8,3]]\},"issued":\{"date-parts":[["2013",5,28]]\},"citation-key":"Chen2013initio"\},"label":"page"\},\{"id":226,"uris":["http://zotero.org/users/9173730/items/KETZF7YZ"],"itemData":\{"id":226,"type":"article-journal","abstract":"Abstract\backslash n We propose a general formalism beyond Weisskopf-Wigner approximation to efficiently calculate the coupling matrix element, evolution spectrum and population evolution of two quantum emitters in arbitrary metallic nanostructures. We demonstrate this formalism to investigate the radiative coupling and decay dynamics of two quantum emitters embedded in the two hot spots of three silver nano-spheroids. The vacuum Rabi oscillation in population evolution and the anti-crossing behavior in evolution spectrum show strong radiative coupling is realized in this metallic nanostructure despite its strong plasmon damping. Our formalism can serve as a flexible and efficient calculation tool to investigate the distant coherent interaction in a large variety of metallic nanostructures, and may be further developed to handle the cases for multiple quantum emitters and arbitrary dielectric-metallic hybrid nanostructures.","container-title":"Scientific Reports","DOI":"10.1038/s41598-022-10624-y","ISSN":"2045-2322","issue":"1","journalAbbreviation":"Sci Rep","language":"en","page":"6901","source":"DOI.org (Crossref)","title":"Radiative coupling of two quantum emitters in arbitrary metallic nanostructures","URL":"https://www.nature.com/articles/s41598-022-10624-y","volume":"12","author":[\{"family":"Liu","given":"JingFeng"\},\{"family":"Chen","given":"Gengyan"\},\{"family":"Li","given":"Lingyan"\},\{"family":"Liu","given":"Renming"\},\{"family":"Li","given":"Wei"\},\{"family":"Liu","given":"Guanghui"\},\{"family":"Wu","given":"Feng"\},\{"family":"Chen","given":"Yongzhu"\}],"accessed":\{"date-parts":[["2022",7,6]]\},"issued":\{"date-parts":[["2022",12]]\},"citation-key":"Liu2022Radiative"\},"label":"act"\},\{"id":238,"uris":["http://zotero.org/users/9173730/items/89I48UEW"],"itemData":\{"id":238,"type":"article-journal","container-title":"Physical Review A","DOI":"10.1103/PhysRevA.70.053823","ISSN":"1050-2947, 1094-1622","issue":"5","journalAbbreviation":"Phys. Rev. A","language":"en","page":"053823","source":"DOI.org (Crossref)","title":"Multiple-scattering approach to interatomic interactions and superradiance in inhomogeneous dielectrics","URL":"https://link.aps.org/doi/10.1103/PhysRevA.70.053823","volume":"70","author":[\{"family":"Wubs","given":"Martijn"\},\{"family":"Suttorp","given":"L. G."\},\{"family":"Lagendijk","given":"A."\}],"accessed":\{"date-parts":[["2022",7,13]]\},"issued":\{"date-parts":[["2004",11,29]]\},"citation-key":"Wubs2004Multiplescattering"\},"label":"act"\}],"schema":"https://github.com/citation-style-language/schema/raw/master/csl-citation.json"\}$^6–8^. Here, the complex frequencies $\varpi^{\pm}=\varpi\pm i\eta$ are assumed to contain a real frequency $\varpi$ and an infinitely small positive and negative imaginary part $\pm i\eta\left( \eta\to0 \right)$. Therefore, the above Eqns. (S4a) - (S4b) under forward Fourier transform $\varpi^{+}=\varpi+i\eta\left( \eta\to0 \right)$ are expressed as

$$\begin{aligned} -C_{e0}-i\varpi C_{e}^{+}\left( \varpi\right)&=-i\omega_{0}C_{e}^{+}\left( \varpi\right) \\ &-\frac{1}{\sqrt{\hbar\pi\epsilon_{0}}}\int_{0}^{\infty} \text{d}\omega\frac{\omega^{2}}{c^{2}}\int\boldsymbol{r}^{\boldsymbol{'}}d\boldsymbol{r}^{\boldsymbol{'}}\sqrt{\epsilon_{I}\left( \boldsymbol{r}^{\boldsymbol{'}},\omega\right)}\mathbf{d}\cdot\mathbf{G}\left( \boldsymbol{r},\boldsymbol{r}^{'},\omega\right)C_{g}^{+}\left( \boldsymbol{r},\omega,\varpi\right),\#\left( S5a \right) \\ -i\varpi C_{g}^{+}\left( \boldsymbol{r},\omega,\varpi\right)&=-i\omega C_{g}^{+}\left( \boldsymbol{r},\omega,\varpi\right) \\ &+C_{e}^{+}\left( \varpi\right)\frac{1}{\sqrt{\hbar\pi\epsilon_{0}}}\int_{0}^{\infty} \text{d}\omega\frac{\omega^{2}}{c^{2}}\sqrt{\epsilon_{I}\left( \boldsymbol{r}^{\boldsymbol{'}},\omega\right)}\mathbf{d}\cdot\mathbf{G}^{\mathbf{*}}\left( \boldsymbol{r},\boldsymbol{r}^{'},\omega\right).\#\left( S5b \right) \end{aligned}$$

To simplify the equations of probability amplitudes, an important relation Eqn. (S6) is introduced as^3^

$$\begin{aligned} \int\frac{\omega^{2}}{c^{2}}\epsilon_{I}\left( \boldsymbol{r}^{\boldsymbol{'}},\omega\right)\mathbf{G}\left( \boldsymbol{r},\boldsymbol{r}^{'},\omega\right)\mathbf{G}^{\mathbf{*}}\left( \boldsymbol{r}^{'},\boldsymbol{r},\omega\right)d\boldsymbol{r}^{\boldsymbol{'}}=\mathrm{Im}\left[ \mathbf{G}\left( \boldsymbol{r},\boldsymbol{r},\omega\right) \right].\#\left( S6 \right) \end{aligned}$$

Moreover, Eqn. S5b substitutes into Eqn. S5a, and it can be written as

$$\begin{aligned} -i\left( \varpi-\omega_{0} \right)C_{e}^{+}\left( \omega^{+} \right)&=C_{e0} \\ &-C_{e}^{+}\left( \varpi\right)\frac{i}{\hbar{\pi\epsilon}_{0}}\int_{0}^{\infty} d\omega\frac{\omega^{2}}{c^{2}\left( \varpi-\omega\right)}\mathbf{d}\cdot Im\left[ \mathbf{G}\left( \boldsymbol{r},\boldsymbol{r},\omega\right) \right]\cdot\mathbf{d}.\#\left( S7 \right) \end{aligned}$$

To further simplify the equation, a significant formula is often used to deal with quantum optics problems. It can be expressed as^9^

$$\begin{aligned} \lim_{\eta\to0^{+}} \int_{0}^{\infty} d\omega\frac{\mathbf{d}\cdot Im\left[ \mathbf{G}\left( \boldsymbol{r},\boldsymbol{r},\omega\right) \right]\cdot\mathbf{d}}{\omega-\varpi+i\eta}=\pi\mathbf{d}\cdot\mathbf{G}\left( \boldsymbol{r},\boldsymbol{r},\varpi\right)\cdot\mathbf{d.}\#\left( S8 \right) \end{aligned}$$

Therefore, we use $\omega$ to replace $\varpi$ , and the forward spectral equation can be written as

$$\begin{aligned} {C_{e}}^{+}\left( \omega\right)=\frac{iC_{e0}}{\omega-\omega_{0}+\frac{\omega^{2}}{\hbar\epsilon_{0}c^{2}}\mathbf{d}\cdot\mathbf{G}\left( \boldsymbol{r},\boldsymbol{r},\omega\right)\cdot\mathbf{d}}.\#\left( S9 \right) \end{aligned}$$

Moreover, we consider a relation that is $-\frac{\omega^{2}}{\hbar\epsilon_{0}c^{2}}\mathbf{d}\cdot\mathbf{G}\left( \boldsymbol{r},\boldsymbol{r},\omega\right)\cdot\mathbf{d}=\Delta\left( \omega\right)- {i\Gamma\left( \omega\right)}/2$, where $\Delta\left( \omega\right)= -\frac{\omega^{2}}{\hbar\epsilon_{0}c^{2}}\mathbf{d}\cdot Re\left[ \mathbf{G}\left( \boldsymbol{r},\boldsymbol{r},\omega\right) \right]\cdot\mathbf{d}$ and $\Gamma\left( \omega\right)= \frac{2\omega^{2}}{\hbar\epsilon_{0}c^{2}}\mathbf{d}\cdot Im\left[ \mathbf{G}\left( \boldsymbol{r},\boldsymbol{r},\omega\right) \right]\cdot\mathbf{d}$, respectively, and they are satisfied the Kramers–Kronig relation. According to the same process, we obtain the backward spectral equation $C_{e}^{-}\left( \omega\right)$ . So that the exactly spectral equation $C_{e}\left( \omega\right)=\left[ C_{e}^{+}\left( \omega\right)+C_{e}^{-}\left( \omega\right) \right]$ can be expressed as^7^

$$\begin{aligned} C_{e}\left( \omega\right)=\frac{1}{\pi}\frac{C_{e0}\cdot\Gamma\left( \omega\right)/2}{\left( \omega-\omega_{0}^{'} \right)^{2}+\left[ \Gamma\left( \omega\right)/2 \right]^{2}},\#\left( S10 \right) \end{aligned}$$

where $\omega_{0}^{'}=\omega_{0}- \Delta\left( \omega\right)$ is that emission frequency considers the shift induced by the nanophotonic structure. Finally, the temporal evolution of the probability amplitude can be calculated by Fourier transform $C_{e}\left( t \right)=\int_{-\infty}^{+\infty} C_{e}\left( \omega\right)e^{-i\omega t} d\omega$.

1. **Derivation** of Entanglement Dynamics

Furthermore, the quantum nanophotonic system, consisting of a pair of QEs ($\mathrm{QE}_{a}$ and $\mathrm{QE}_{b}$) and nanophotonic structure, can generate entanglement between the QEs. Similarly, the entanglement dynamics can be represented by the excited-state population $\left| C_{a,b}(t) \right|$, so that we deduce a formalism to calculate the temporal evolution of the probability amplitudes $C_{a,b}(t)$ in the quantum nanophotonic system. The Hamiltonian of the quantum nanophotonic system can be expressed as

$$\begin{aligned} \hat{\boldsymbol{H}}&=\int d^{3}\boldsymbol{r}\int_{0}^{\infty} \hbar\omega{\hat{\boldsymbol{f}}}^{+}\left( \boldsymbol{r},\omega\right)\hat{\boldsymbol{f}}\left( \boldsymbol{r},\omega\right)d\omega+\sum_{m=a, b} \hbar\omega_{m}\left| e_{m} \right\rangle\left\langle e_{m} \right| \\ &+\sum_{m=a, b} \int_{0}^{\infty} d\omega\left[ \sigma_{m}^{+}\mathbf{d}_{m}\cdot{\hat{\mathbf{E}}}^{+}\left( \boldsymbol{r}_{m},\omega\right)+H.c. \right].\#\left( S11 \right) \end{aligned}$$

Here, the first term also represents the field energy of the environment in the presence of the nanophotonic structure. $\hat{\boldsymbol{f}}\left( \boldsymbol{r},\omega\right)$ and ${\hat{\boldsymbol{f}}}^{+}\left( \boldsymbol{r},\omega\right)$ are the bosonic vector field annihilation and creation operators for the elementary excitations of the system, respectively. $\omega_{m}$ and $\mathbf{d}_{m}$ are the transition frequency and transition dipole moment between the excited state $\left| e_{m} \right\rangle$ and the ground state $\left| g_{m} \right\rangle$ of the $m^{th}\left( m=a,b \right)$ QE located at $\boldsymbol{r}_{m}$. In addition, the time evaluation can be expressed as

$$\begin{aligned} \left| \Psi\left( t \right) \right\rangle&=C_{a}\left( t \right)\left| e_{a},g_{b} \right\rangle\left| 0 \right\rangle+C_{b}\left( t \right)\left| g_{a},e_{b} \right\rangle\left| 0 \right\rangle\\ &+\int d^{3}\boldsymbol{r}\int_{0}^{\infty} d\omega C_{g}\left( \boldsymbol{r},\omega,t \right)\left| g_{a},g_{b} \right\rangle\left| \mathbf{1}\left( \boldsymbol{r},\omega\right) \right\rangle,\#\left( S12 \right) \end{aligned}$$

where, $\left| 0 \right\rangle$ is the vacuum state of the electromagnetic fields, and $\left| \mathbf{1}\left( \boldsymbol{r},\omega\right) \right\rangle$ is the single-quantum Fock state of the fields. The initial conditions are $C_{a0}=1$, $C_{b0}=0$, and $C_{g0}=0$. The time-dependent Schrodinger equation with the orthogonal and complete properties leads to the following equation of motion for the probability amplitudes:

$$\begin{aligned} \dot{C_{a}}\left( t \right)&=-i\omega_{a}C_{a}\left( t \right) \\ &-\frac{1}{\sqrt{\hbar\pi\epsilon_{0}}}\int_{0}^{\infty} \text{d}\omega\int d\boldsymbol{r}^{\boldsymbol{'}}\frac{\omega^{2}}{c^{2}}\sqrt{\epsilon_{I}\left( \boldsymbol{r}^{\boldsymbol{'}},\omega\right)}\mathbf{d}_{a}\cdot\mathbf{G}\left( \boldsymbol{r}_{a},\boldsymbol{r}^{'},\omega\right)C_{g}\left( \boldsymbol{r}^{\boldsymbol{'}},\omega,t \right),\#\left( S13a \right) \\ \dot{C_{b}}\left( t \right)&=-i\omega_{b}C_{b}\left( t \right) \\ &-\frac{1}{\sqrt{\hbar\pi\epsilon_{0}}}\int_{0}^{\infty} \text{d}\omega\int d\boldsymbol{r}^{\boldsymbol{'}}\frac{\omega^{2}}{c^{2}}\sqrt{\epsilon_{I}\left( \boldsymbol{r}^{\boldsymbol{'}},\omega\right)}\mathbf{d}_{b}\cdot\mathbf{G}\left( \boldsymbol{r}_{b},\boldsymbol{r}^{'},\omega\right)C_{g}\left( \boldsymbol{r}^{\boldsymbol{'}},\omega,t \right),\#\left( S13b \right) \\ \dot{C_{g}}\left( \boldsymbol{r}^{'},\omega,t \right)&=-i\omega C_{g}\left( \boldsymbol{r}^{\boldsymbol{'}},\omega,t \right) \\ &+\sum_{m=a,b} C_{m}\left( t \right)\frac{1}{\sqrt{\hbar\pi\epsilon_{0}}}\frac{\omega^{2}}{c^{2}}\sqrt{\epsilon_{I}\left( \boldsymbol{r}^{\boldsymbol{'}},\omega\right)}\mathbf{d}_{m}\cdot\mathbf{G}^{\mathbf{*}}\left( \boldsymbol{r}_{m},\boldsymbol{r}^{'},\omega\right).\#\left( S13c \right) \end{aligned}$$

Likewise, we do forward Fourier transform $\varpi^{+}=\varpi+i\eta\left( \eta\to0 \right)$ for the above equations as that do in Eqns. (S5a) - (S5b):

$$\begin{aligned} -i\varpi C_{a}^{+}\left( \varpi\right)&=C_{a0}-i\omega_{a}C_{a}^{+}\left( \varpi\right) \\ &-\frac{1}{\sqrt{\hbar\pi\epsilon_{0}}}\int_{0}^{\infty} \text{d}\omega\int d\boldsymbol{r}^{\boldsymbol{'}}\frac{\omega^{2}}{c^{2}}\sqrt{\epsilon_{I}\left( \boldsymbol{r}^{\boldsymbol{'}},\omega\right)}\mathbf{d}_{a}\cdot\mathbf{G}\left( \boldsymbol{r}_{a},\boldsymbol{r}^{'},\omega\right)C_{g}^{+}\left( \boldsymbol{r}^{\boldsymbol{'}},\omega,\varpi\right),\#\left( S14a \right) \\ -i\varpi C_{b}^{+}\left( \varpi\right)&=C_{b0}-i\omega_{b}C_{b}^{+}\left( \varpi\right) \\ &-\frac{1}{\sqrt{\hbar\pi\epsilon_{0}}}\int_{0}^{\infty} \text{d}\omega\int d\boldsymbol{r}^{\boldsymbol{'}}\frac{\omega^{2}}{c^{2}}\sqrt{\epsilon_{I}\left( \boldsymbol{r}^{\boldsymbol{'}},\omega\right)}\mathbf{d}_{b}\cdot\mathbf{G}\left( \boldsymbol{r}_{b},\boldsymbol{r}^{'},\omega\right)C_{g}^{+}\left( \boldsymbol{r}^{\boldsymbol{'}},\omega,\varpi\right),\#\left( S14b \right) \\ -i\varpi C_{g}^{+}\left( \boldsymbol{r}^{\boldsymbol{'}},\omega,\varpi\right)&=-i\varpi C_{g}^{+}\left( \boldsymbol{r}^{\boldsymbol{'}},\omega,\varpi\right) \\ &+\sum_{m=a,b} C_{m}\left( \varpi\right)\frac{1}{\sqrt{\hbar\pi\epsilon_{0}}}\frac{\omega^{2}}{c^{2}}\sqrt{\epsilon_{I}\left( \boldsymbol{r}^{\boldsymbol{'}},\omega\right)}\mathbf{d}_{m}\cdot\mathbf{G}^{\mathbf{*}}\left( \boldsymbol{r}_{m},\boldsymbol{r}^{'},\omega\right).\#\left( S14c \right) \end{aligned}$$

With the important relation Eqn. (S6), and Eqn. (S14c) substituting into Eqn. (S14a) - (S14b), we can simplify the equations of probability amplitudes:

$$\begin{aligned} -i\left[ \left( \varpi-\omega_{a} \right)-\frac{1}{\hbar{\pi\epsilon}_{0}}\int_{0}^{\infty} d\omega\frac{\omega^{2}}{c^{2}\left( \varpi-\omega\right)}\mathbf{d}_{a}\cdot Im\left[ \mathbf{G}\left( \boldsymbol{r}_{a},\boldsymbol{r}_{a},\omega\right) \right]\cdot\mathbf{d}_{a} \right]C_{a}^{+}\left( \varpi\right)=C_{a0} \\ &-\frac{i}{\hbar{\pi\epsilon}_{0}}\int_{0}^{\infty} d\omega\frac{\omega^{2}}{c^{2}\left( \varpi-\omega\right)}\mathbf{d}_{a}\cdot Im\left[ \mathbf{G}\left( \boldsymbol{r}_{a},\boldsymbol{r}_{b},\omega\right) \right]\cdot\mathbf{d}_{b}C_{b}^{+}\left( \varpi\right),\#\left( S15a \right) \\ -i\left[ \left( \varpi-\omega_{b} \right)-\frac{1}{\hbar{\pi\epsilon}_{0}}\int_{0}^{\infty} d\omega\frac{\omega^{2}}{c^{2}\left( \varpi-\omega\right)}\mathbf{d}_{b}\cdot Im\left[ \mathbf{G}\left( \boldsymbol{r}_{b},\boldsymbol{r}_{b},\omega\right) \right]\cdot\mathbf{d}_{b} \right]C_{b}^{+}\left( \varpi\right)=C_{b0} \\ -\frac{i}{\hbar{\pi\epsilon}_{0}}\int_{0}^{\infty} d\omega\frac{\omega^{2}}{c^{2}\left( \varpi-\omega\right)}\mathbf{d}_{b}\cdot Im\left[ \mathbf{G}\left( \boldsymbol{r}_{b},\boldsymbol{r}_{a},\omega\right) \right]\cdot\mathbf{d}_{a}C_{a}^{+}\left( \varpi\right).\#\left( S15b \right) \end{aligned}$$

With the significant formula Eqn. (S8) the equations can further simply. Moreover, we use $\omega$ to replace $\varpi$, and the exactly spectral equation $C_{m}\left( \omega\right)=\left[ C_{m}^{+}\left( \omega\right)+C_{m}^{-}\left( \omega\right) \right]$ can be written as^8^

$$\begin{aligned} C_{a}\left( \omega\right)=2Re\left\{ \frac{i\left[ \left( \omega-\omega_{b}+\frac{i\Gamma_{bb}\left( \omega\right)}{2} \right)C_{a0}+\left( \Delta_{ab}\left( \omega\right)-\frac{i\Gamma_{ab}\left( \omega\right)}{2} \right)C_{b0} \right]}{\left( \omega-\omega_{a}+\frac{i\Gamma_{aa}\left( \omega\right)}{2} \right)\left( \omega-\omega_{b}+\frac{i\Gamma_{bb}\left( \omega\right)}{2} \right)-\left( \Delta_{ab}\left( \omega\right)-\frac{i\Gamma_{ab}\left( \omega\right)}{2} \right)^{2}} \right\},\#\left( S16a \right) \end{aligned}$$

$$\begin{aligned} C_{b}\left( \omega\right)=2Re\left\{ \frac{i\left[ \left( \omega-\omega_{a}+\frac{i\Gamma_{aa}\left( \omega\right)}{2} \right)C_{b0}+\left( \Delta_{ab}\left( \omega\right)-\frac{i\Gamma_{ab}\left( \omega\right)}{2} \right)C_{a0} \right]}{\left( \omega-\omega_{a}^{'}+\frac{i\Gamma_{aa}\left( \omega\right)}{2} \right)\left( \omega-\omega_{b}^{'}+\frac{i\Gamma_{bb}\left( \omega\right)}{2} \right)-\left( \Delta_{ab}\left( \omega\right)-\frac{i\Gamma_{ab}\left( \omega\right)}{2} \right)^{2}} \right\},\#\left( S16b \right) \end{aligned}$$

where, $\Delta_{mn}\left( \omega\right)=-\frac{\omega^{2}}{\hbar\epsilon_{0}c^{2}}\mathbf{d}_{m}\cdot Re\left[ \mathbf{G}\left( \boldsymbol{r}_{m},\boldsymbol{r}_{n},\omega\right) \right]\cdot\mathbf{d}_{m}$, $\Gamma_{mn}\left( \omega\right)=\frac{2\omega^{2}}{\hbar\epsilon_{0}c^{2}}\mathbf{d}_{a}\cdot Im\left[ \mathbf{G}\left( \boldsymbol{r}_{m},\boldsymbol{r}_{n},\omega\right) \right]\cdot\mathbf{d}_{n}$, $\left( m,n=a \mathrm{or} b \right)$, they are satisfied the Kramers–Kronig relation. Specially, $\Delta_{ab}\left( \omega\right)=\Delta_{ba}\left( \omega\right)$ and $\Gamma_{ab}\left( \omega\right)=\Gamma_{ba}\left( \omega\right)$. And $\omega_{m}^{'}=\omega_{m}-\Delta_{mm}\left( \omega\right)$ is that emission frequency considers the shift induced by the nanophotonic structure. Finally, the temporal evolution of the probability amplitude can be calculated by Fourier transform $C\left( t \right)=\frac{1}{2\pi}\int_{-\infty}^{+\infty} C\left( \omega\right)e^{-i\omega t} d\omega$.

1. Analytical Solution of Green's Function for Multilayer Nanoparticle

The dyadic Green’s function for multilayer spherical nanoparticle have received widely research. Starting with the Maxwell’s equations, the electromagnetic fields $\boldsymbol{E}_{f}$and $\boldsymbol{H}_{f}$ in the $f$th layer ($f = 1, 2, ....,N$), contributed by the electric and magnetic current sources $\boldsymbol{J}_{s}$ and $\boldsymbol{M}_{s}$ located in the $s$th layer ($s = 1, 2, ....,N$), of a spherically $N$ - layered medium can be expressed as^10^

$$\begin{aligned} \nabla\times\nabla\times\mathbf{E}_{f}-k_{f}^{2}\mathbf{E}_{f}=i\omega\mu_{f}\boldsymbol{J}_{f}\delta_{f}^{s}-\left( \nabla\times\mathbf{M} \right)_{f}\delta_{f}^{s}, \#\left( S17a \right) \end{aligned}$$

$$\begin{aligned} \nabla\times\nabla\times\mathbf{H}_{f}-k_{f}^{2}\mathbf{H}_{f}=i\omega\mu_{f}\boldsymbol{M}_{f}\delta_{f}^{s}+\left( \nabla\times\boldsymbol{J} \right)_{f}\delta_{f}^{s}, \#\left( S17b \right) \end{aligned}$$

where $k_{f}$in the $f$th layer of the multilayered medium is derived as $k_{f}=\frac{\omega}{c}\sqrt{\mu_{f}\varepsilon_{f}\left( 1+\frac{i\sigma_{f}}{\omega\varepsilon_{f}} \right)}$ with $\sigma_{f}$ is the conductivity of the medium, and $\delta_{f}^{s}$ denotes the Kronecker delta. Solving Eqns. (S17a) and (S17b) yield, respectively^10^

$$\begin{aligned} \nabla\times\nabla\times\boldsymbol{G}_{e}^{\left( fs \right)}\left( \boldsymbol{r},\boldsymbol{r}^{'} \right)-k_{f}^{2}\boldsymbol{G}_{e}^{\left( fs \right)}\left( \boldsymbol{r},\boldsymbol{r}^{'} \right)=\boldsymbol{I}\delta\left( \boldsymbol{r}-\boldsymbol{r}^{'} \right)\delta_{f}^{s}, \#\left( S18a \right) \end{aligned}$$

$$\begin{aligned} \nabla\times\nabla\times\boldsymbol{G}_{m}^{\left( fs \right)}\left( \boldsymbol{r},\boldsymbol{r}^{'} \right)-k_{f}^{2}\boldsymbol{G}_{m}^{\left( fs \right)}\left( \boldsymbol{r},\boldsymbol{r}^{'} \right)=\nabla\times\left[ \boldsymbol{I}\delta\left( \boldsymbol{r}-\boldsymbol{r}^{'} \right) \right]\delta_{f}^{s}, \#\left( S18b \right) \end{aligned}$$

where $\boldsymbol{G}_{e}^{\left( fs \right)}\left( \boldsymbol{r},\boldsymbol{r}^{'} \right)$ is the electric dyadic Green’s function, $\boldsymbol{G}_{m}^{\left( fs \right)}\left( \boldsymbol{r},\boldsymbol{r}^{'} \right)$ is the magnetic dyadic Green’s function, and they satisfy the equation

$$\begin{aligned} \nabla\times\boldsymbol{G}_{m}^{\left( fs \right)}\left( \boldsymbol{r},\boldsymbol{r}^{'} \right)=\boldsymbol{I}\delta\left( \boldsymbol{r}-\boldsymbol{r}^{'} \right)+k_{f}^{2}\boldsymbol{G}_{e}^{\left( fs \right)}\left( \boldsymbol{r},\boldsymbol{r}^{'} \right),\#\left( S19 \right) \end{aligned}$$

where$\boldsymbol{I}$ is the unit dyadic and $\delta\left( \boldsymbol{r}-\boldsymbol{r}^{'} \right)$ is the Dirac delta function.

Here, the electromagnetic dyadic Green’s functions can be expressed with the method of vector eigenfunction expansion^11^. Since our model is a multilayer spherical nanoparticle, the spherical vector eigenfunctions which are solutions of Eqns. (S18a) and (S18b) can be constructed as the spherical vector wave functions^10,11^

$$\begin{aligned} \boldsymbol{L}_{\begin{matrix} e \\ o \end{matrix}nm}\left( k,\boldsymbol{r} \right)&=\nabla j_{n}\left( kr \right)Y_{n}^{m}\left( \theta,\phi\right) \\ &=\frac{\partial j_{n}\left( kr \right)}{\partial r}Y_{n}^{m}\left( \theta,\phi\right)\hat{\boldsymbol{r}}+\frac{j_{n}\left( kr \right)}{r}\left[ \frac{\partial Y_{n}^{m}\left( \theta,\phi\right)}{\partial\theta}\hat{\boldsymbol{\theta}}+\frac{1}{\sin\theta}\frac{\partial Y_{n}^{m}\left( \theta,\phi\right)}{\partial\phi}\hat{\boldsymbol{\phi}} \right],\#\left( S20a \right) \end{aligned}$$

$$\begin{aligned} \boldsymbol{M}_{\begin{matrix} e \\ o \end{matrix}nm}\left( \boldsymbol{r},k \right)&=\nabla\times\boldsymbol{r}j_{n}\left( kr \right)Y_{n}^{m}\left( \theta,\phi\right) \\ &=j_{n}\left( kr \right)\left[ \frac{1}{\sin\theta}\frac{\partial Y_{n}^{m}\left( \theta,\phi\right)}{\partial\phi}\hat{\boldsymbol{\theta}}-\frac{\partial Y_{n}^{m}\left( \theta,\phi\right)}{\partial\theta}\hat{\boldsymbol{\phi}} \right],\#\left( S20b \right) \end{aligned}$$

$$\begin{aligned} \boldsymbol{N}_{\begin{matrix} e \\ o \end{matrix}nm}\left( k,\boldsymbol{r} \right)&=\frac{1}{k}\nabla\times\nabla\times\boldsymbol{r}j_{n}\left( kr \right)Y_{n}^{m}\left( \theta,\phi\right) \\ &=-\frac{n\left( n+1 \right)}{kr}j_{n}\left( kr \right)Y_{n}^{m}\left( \theta,\phi\right)\hat{\boldsymbol{r}} \\ &+\frac{1}{kr}\frac{\partial rj_{n}\left( kr \right)}{\partial r}\left[ \frac{\partial Y_{n}^{m}\left( \theta,\phi\right)}{\partial\theta}\hat{\boldsymbol{\theta}}+\frac{1}{\sin\theta}\frac{\partial Y_{n}^{m}\left( \theta,\phi\right)}{\partial\phi}\hat{\boldsymbol{\phi}} \right],\#\left( S20c \right) \end{aligned}$$

where $j_{n}\left( kr \right)$ denotes the spherical Bessel function of *n*-order, and $Y_{n}^{m}\left( \theta,\phi\right)=P_{n}^{\left( m \right)}\left( cos\theta\right)e^{im\phi}$ is the spherical harmonics, in which $P_{n}^{\left( m \right)}\left( cos\theta\right)$ identities the associated Legendre function of the first kind with the order (*n, m*)^10,12^. Using the spherical vector wave functions, we may construct the dyadic Green’s function subsequently. Firstly, the dyadic Green’s function of a homogeneous medium or free pace can be expressed as^10^

$$\begin{aligned} \boldsymbol{G}_{e0}\left( \boldsymbol{r},\boldsymbol{r}^{'} \right)&=-\frac{\delta\left( r-r^{'} \right)}{k_{s}^{2}}\hat{\boldsymbol{r}}\hat{\boldsymbol{r}}+\frac{ik_{s}}{4\pi}\sum_{eo} \sum_{n=1}^{\infty} \sum_{m=0}^{n} \left( 2-\delta_{0m} \right)\frac{2n+1}{n\left( n+1 \right)}\frac{\left( n-m \right)!}{\left( n+m \right)!} \\ &\times\left\{ \begin{matrix} \boldsymbol{M}_{\begin{matrix} e \\ o \end{matrix}nm}^{\left( 1 \right)}\left( k_{s},\boldsymbol{r} \right)\boldsymbol{M}_{\begin{matrix} e \\ o \end{matrix}nm}^{*}\left( k_{s},\boldsymbol{r}^{'} \right)+\boldsymbol{N}_{\begin{matrix} e \\ o \end{matrix}nm}^{\left( 1 \right)}\left( k_{s},\boldsymbol{r} \right)\boldsymbol{N}_{\begin{matrix} e \\ o \end{matrix}nm}^{*}\left( k_{s},\boldsymbol{r}^{'} \right) \left( r>r^{'} \right) \\ \boldsymbol{M}_{\begin{matrix} e \\ o \end{matrix}nm}\left( k_{s},\boldsymbol{r} \right)\boldsymbol{M}_{\begin{matrix} e \\ o \end{matrix}nm}^{*\left( 1 \right)}\left( k_{s},\boldsymbol{r}^{'} \right)+\boldsymbol{N}_{\begin{matrix} e \\ o \end{matrix}nm}\left( k_{s},\boldsymbol{r} \right)\boldsymbol{N}_{\begin{matrix} e \\ o \end{matrix}nm}^{*\left( 1 \right)}\left( k_{s},\boldsymbol{r}^{'} \right) \left( r^{'}>r \right) \end{matrix} \right.,\#\left( S21 \right) \end{aligned}$$

where $k_{s}=\frac{\omega}{c}\sqrt{\varepsilon_{s}}$ can be considered the current source and response field locations both at $s$th layer, the prime denotes the coordinates $\left( r^{'},\theta^{'},\phi^{'} \right)$ of the current source, $n$ and $m$ identify the eigenvalue parameters, and $\boldsymbol{M}_{\begin{matrix} e \\ o \end{matrix}nm}$ stands for the electric field of the TE*_mn_* mode, while $\boldsymbol{N}_{\begin{matrix} e \\ o \end{matrix}nm}$ represents that of the TM*_mn_* mode. The superscript $\left( 1 \right)$ denotes that the third-type spherical Bessel function or the first-type spherical Hankel function $h_{n}^{\left( 1 \right)}\left( r \right)$ should be chosen in the expression of the spherical wave vector functions. For the rest of the vector wave functions, we should still choose the normal first type spherical Bessel function $j_{n}\left( r \right)$ because this type of function can be used to represent both out-going and in-coming waves. Considering a multilayer spherical nanoparticle in the medium, the dyadic Green’s function can be considered as the sum of an unbounded dyadic Green’s function and a scattering dyadic Green’s function, with the method of scattering superposition^13^. The dyadic Green’s function is given by

$$\begin{aligned} \boldsymbol{G}_{e}^{\left( fs \right)}\left( \boldsymbol{r},\boldsymbol{r}^{'} \right)=\boldsymbol{G}_{e0}\left( \boldsymbol{r},\boldsymbol{r}^{'} \right)+\boldsymbol{G}_{es}^{\left( fs \right)}\left( \boldsymbol{r},\boldsymbol{r}^{'} \right),\#\left( S22 \right) \end{aligned}$$

where the scattering dyadic Green’s function $\boldsymbol{G}_{es}^{\left( fs \right)}\left( \boldsymbol{r},\boldsymbol{r}^{'} \right)$ describes an additional contribution of the multiple reflection and transmission waves in the presence of the boundary of dielectric media. Under the spherical coordinates, the electromagnetic fields usually consist of the radial wave modes propagating outwards and inwards. Hence, assuming that the current source is located in the layer $s$th layer ($s = 1, 2, ....,N$), we may construct the scattering dyadic Green’s function for the $f$th layer ($f = 1, 2, ....,N$) among the multilayers with the spherical Bessel and Hankel functions as follows^10^

$$\begin{aligned} \boldsymbol{G}_{es}^{\left( fs \right)}\left( \boldsymbol{r},\boldsymbol{r}^{'} \right)&=\frac{ik_{s}}{4\pi}\sum_{eo} \sum_{n=1}^{\infty} \sum_{m=0}^{n} \left( 2-\delta_{0m} \right)\frac{2n+1}{n\left( n+1 \right)}\frac{\left( n-m \right)!}{\left( n+m \right)!} \\ &\times\{\left( 1-\delta_{f}^{N} \right)\boldsymbol{M}_{\begin{matrix} e \\ o \end{matrix}nm}^{\left( 1 \right)}\left( k_{f},\boldsymbol{r} \right)\left[ \left( 1-\delta_{s}^{1} \right)A_{\boldsymbol{M}}^{fs}\boldsymbol{M}_{\begin{matrix} e \\ o \end{matrix}nm}^{*}\left( k_{s},\boldsymbol{r}^{'} \right)+\left( 1-\delta_{s}^{N} \right)B_{\boldsymbol{M}}^{fs}\boldsymbol{M}_{\begin{matrix} e \\ o \end{matrix}nm}^{*\left( 1 \right)}\left( k_{s},\boldsymbol{r}^{'} \right) \right] \\ &+\left( 1-\delta_{f}^{N} \right)\boldsymbol{N}_{\begin{matrix} e \\ o \end{matrix}nm}^{\left( 1 \right)}\left( k_{f},\boldsymbol{r} \right)\left[ \left( 1-\delta_{s}^{1} \right)A_{\boldsymbol{N}}^{fs}\boldsymbol{N}_{\begin{matrix} e \\ o \end{matrix}nm}^{*}\left( k_{s},\boldsymbol{r}^{'} \right)+\left( 1-\delta_{s}^{N} \right)B_{\boldsymbol{N}}^{fs}\boldsymbol{N}_{\begin{matrix} e \\ o \end{matrix}nm}^{*\left( 1 \right)}\left( k_{s},\boldsymbol{r}^{'} \right) \right] \\ &+ \left( 1-\delta_{f}^{1} \right)\boldsymbol{M}_{\begin{matrix} e \\ o \end{matrix}nm}\left( k_{f},\boldsymbol{r} \right)\left[ \left( 1-\delta_{s}^{1} \right)C_{\boldsymbol{M}}^{fs}\boldsymbol{M}_{\begin{matrix} e \\ o \end{matrix}nm}^{*}\left( k_{s},\boldsymbol{r}^{'} \right)+\left( 1-\delta_{s}^{N} \right)D_{\boldsymbol{M}}^{fs}\boldsymbol{M}_{\begin{matrix} e \\ o \end{matrix}nm}^{*\left( 1 \right)}\left( k_{s},\boldsymbol{r}^{'} \right) \right] \\ &+\left( 1-\delta_{f}^{1} \right)\boldsymbol{N}_{\begin{matrix} e \\ o \end{matrix}nm}\left( k_{f},\boldsymbol{r} \right)\left[ \left( 1-\delta_{s}^{1} \right)C_{\boldsymbol{N}}^{fs}\boldsymbol{N}_{\begin{matrix} e \\ o \end{matrix}nm}^{*}\left( k_{s},\boldsymbol{r}^{'} \right)+\left( 1-\delta_{s}^{N} \right)D_{\boldsymbol{N}}^{fs}\boldsymbol{N}_{\begin{matrix} e \\ o \end{matrix}nm}^{*\left( 1 \right)}\left( k_{s},\boldsymbol{r}^{'} \right) \right]\}, \\ \\ \left( S23 \right) \end{aligned}$$

where, $A_{\boldsymbol{M,N}}^{fs}$, $B_{\boldsymbol{M,N}}^{fs}$, $C_{\boldsymbol{M,N}}^{fs}$ and $D_{\boldsymbol{M,N}}^{fs}$ are the coefficients of the scattering dyadic Green’s function to be solved. These coefficients are determined by the boundary conditions at the spherical interfaces^10^

$$\begin{aligned} \boldsymbol{r\times}\boldsymbol{G}_{e}^{\left( fs \right)}\left( \boldsymbol{r},\boldsymbol{r}^{'} \right)=\boldsymbol{r\times}\boldsymbol{G}_{e}^{\left[ \left( f+1 \right)s \right]}\left( \boldsymbol{r},\boldsymbol{r}^{'} \right)\boldsymbol{,\#}\left( S24a \right) \end{aligned}$$

$$\begin{aligned} \frac{1}{\mu_{f}}\boldsymbol{r\times}\boldsymbol{\nabla}\boldsymbol{\times}\boldsymbol{G}_{e}^{\left( fs \right)}\left( \boldsymbol{r},\boldsymbol{r}^{'} \right)=\frac{1}{\mu_{f+1}}\boldsymbol{r\times}\boldsymbol{\nabla}\boldsymbol{\times}\boldsymbol{G}_{e}^{\left[ \left( f+1 \right)s \right]}\left( \boldsymbol{r},\boldsymbol{r}^{'} \right)\boldsymbol{.\#}\left( S24b \right) \end{aligned}$$

In our case, we set the current source and response field locations both at the $s=f=1$st layer, so that the scattering dyadic Green’s function can be simplified as

$$\begin{aligned} \boldsymbol{G}_{es}^{\left( 11 \right)}\left( \boldsymbol{r},\boldsymbol{r}^{'} \right)&=\frac{ik_{1}}{4\pi}\sum_{eo} \sum_{n=1}^{\infty} \sum_{m=0}^{n} \left( 2-\delta_{0m} \right)\frac{2n+1}{n\left( n+1 \right)}\frac{\left( n-m \right)!}{\left( n+m \right)!} \\ &\times\left( B_{\boldsymbol{M}}^{11}\boldsymbol{M}_{\begin{matrix} e \\ o \end{matrix}nm}^{\left( 1 \right)}\left( k_{1},\boldsymbol{r} \right)\boldsymbol{M}_{\begin{matrix} e \\ o \end{matrix}nm}^{*\left( 1 \right)}\left( k_{1},\boldsymbol{r}^{'} \right)+B_{\boldsymbol{N}}^{11}\boldsymbol{N}_{\begin{matrix} e \\ o \end{matrix}nm}^{\left( 1 \right)}\left( k_{1},\boldsymbol{r} \right)\boldsymbol{N}_{\begin{matrix} e \\ o \end{matrix}nm}^{*\left( 1 \right)}\left( k_{1},\boldsymbol{r}^{'} \right) \right)\boldsymbol{.}\boldsymbol{\#}\left（ S25 \right） \end{aligned}$$

Therefore, we only need to get the coefficients $B_{\boldsymbol{M,N}}^{11}$ of the scattering dyadic Green’s function. With the boundary conditions at the spherical interfaces, we also need the scattering dyadic Green’s function at the other $f$th layer

$$\begin{aligned} \boldsymbol{G}_{es}^{\left( f1 \right)}\left( \boldsymbol{r},\boldsymbol{r}^{'} \right)&=\frac{ik_{1}}{4\pi}\sum_{eo} \sum_{n=1}^{\infty} \sum_{m=0}^{n} \left( 2-\delta_{0m} \right)\frac{2n+1}{n\left( n+1 \right)}\frac{\left( n-m \right)!}{\left( n+m \right)!} \\ &\times\{B_{\boldsymbol{M}}^{f1}\boldsymbol{M}_{\begin{matrix} e \\ o \end{matrix}nm}^{\left( 1 \right)}\left( k_{f},\boldsymbol{r} \right)\boldsymbol{M}_{\begin{matrix} e \\ o \end{matrix}nm}^{*\left( 1 \right)}\left( k_{1},\boldsymbol{r}^{'} \right)+B_{\boldsymbol{N}}^{f1}\boldsymbol{N}_{\begin{matrix} e \\ o \end{matrix}nm}^{\left( 1 \right)}\left( k_{f},\boldsymbol{r} \right)\boldsymbol{N}_{\begin{matrix} e \\ o \end{matrix}nm}^{*\left( 1 \right)}\left( k_{1},\boldsymbol{r}^{'} \right) \\ &+ D_{\boldsymbol{M}}^{f1}\boldsymbol{M}_{\begin{matrix} e \\ o \end{matrix}nm}\left( k_{f},\boldsymbol{r} \right)\boldsymbol{M}_{\begin{matrix} e \\ o \end{matrix}nm}^{*\left( 1 \right)}\left( k_{1},\boldsymbol{r}^{'} \right)+D_{\boldsymbol{N}}^{f1}\boldsymbol{N}_{\begin{matrix} e \\ o \end{matrix}nm}\left( k_{f},\boldsymbol{r} \right)\boldsymbol{N}_{\begin{matrix} e \\ o \end{matrix}nm}^{*\left( 1 \right)}\left( k_{1},\boldsymbol{r}^{'} \right)\}, \left( f\neq1 \right)\boldsymbol{.}\boldsymbol{\#}\left（ S26 \right） \end{aligned}$$

Here, if $f=N$th layer, the scattering dyadic Green’s function can be rewritten as

$$\begin{aligned} \boldsymbol{G}_{es}^{\left( N1 \right)}\left( \boldsymbol{r},\boldsymbol{r}^{'} \right)&=\frac{ik_{1}}{4\pi}\sum_{eo} \sum_{n=1}^{\infty} \sum_{m=0}^{n} \left( 2-\delta_{0m} \right)\frac{2n+1}{n\left( n+1 \right)}\frac{\left( n-m \right)!}{\left( n+m \right)!} \\ &\times\left( D_{\boldsymbol{M}}^{N1}\boldsymbol{M}_{\begin{matrix} e \\ o \end{matrix}nm}\left( k_{f},\boldsymbol{r} \right)\boldsymbol{M}_{\begin{matrix} e \\ o \end{matrix}nm}^{*\left( 1 \right)}\left( k_{1},\boldsymbol{r}^{'} \right)+D_{\boldsymbol{N}}^{N1}\boldsymbol{N}_{\begin{matrix} e \\ o \end{matrix}nm}\left( k_{f},\boldsymbol{r} \right)\boldsymbol{N}_{\begin{matrix} e \\ o \end{matrix}nm}^{*\left( 1 \right)}\left( k_{1},\boldsymbol{r}^{'} \right) \right)\boldsymbol{.}\boldsymbol{\#}\left（ S27 \right） \end{aligned}$$

Combining the boundary conditions and the expression of the dyadic Green’s function, the boundary conditions can be rewritten as the following coefficients matrix equations:

$$\begin{aligned} \left[ \begin{matrix} h_{ff}^{\left( 1 \right)} & j_{ff} \\ \frac{k_{f}}{\mu_{f}}\partial h_{ff}^{\left( 1 \right)} & \frac{k_{f}}{\mu_{f}}\partial j_{ff} \end{matrix} \right]\left[ \begin{matrix} B_{\boldsymbol{M}}^{f1} \\ \left( D_{\boldsymbol{M}}^{f1}+\delta_{f}^{1} \right) \end{matrix} \right]=\left[ \begin{matrix} h_{\left( f+1 \right)f}^{\left( 1 \right)} & j_{\left( f+1 \right)f} \\ \frac{k_{f+1}}{\mu_{f+1}}\partial h_{\left( f+1 \right)f}^{\left( 1 \right)} & \frac{k_{f+1}}{\mu_{f+1}}\partial j_{\left( f+1 \right)f} \end{matrix} \right]\left[ \begin{matrix} B_{\boldsymbol{M}}^{\left( f+1 \right)1} \\ D_{\boldsymbol{M}}^{\left( f+1 \right)1} \end{matrix} \right],\#\left( S28a \right) \end{aligned}$$

$$\begin{aligned} \left[ \begin{matrix} \partial h_{ff}^{\left( 1 \right)} & \partial j_{ff} \\ \frac{k_{f}}{\mu_{f}}h_{ff}^{\left( 1 \right)} & \frac{k_{f}}{\mu_{f}}j_{ff} \end{matrix} \right]\left[ \begin{matrix} B_{\boldsymbol{N}}^{f1} \\ \left( D_{\boldsymbol{N}}^{f1}+\delta_{f}^{1} \right) \end{matrix} \right]=\left[ \begin{matrix} \partial h_{\left( f+1 \right)f}^{\left( 1 \right)} & \partial j_{\left( f+1 \right)f} \\ \frac{k_{f+1}}{\mu_{f+1}}h_{\left( f+1 \right)f}^{\left( 1 \right)} & \frac{k_{f+1}}{\mu_{f+1}}j_{\left( f+1 \right)f} \end{matrix} \right]\left[ \begin{matrix} B_{\boldsymbol{N}}^{\left( f+1 \right)1} \\ D_{\boldsymbol{N}}^{\left( f+1 \right)1} \end{matrix} \right],\#\left( S28b \right) \end{aligned}$$

where $h_{il}^{\left( 1 \right)}=h_{n}^{\left( 1 \right)}\left( \rho_{il} \right)$, $j_{il}=j_{n}\left( \rho_{il} \right)$, $\partial h_{il}^{\left( 1 \right)}=\frac{1}{\rho_{il}}\frac{\partial\rho_{il}h_{n}^{\left( 1 \right)}\left( \rho_{il} \right)}{\partial\rho_{il}}$ and $\partial j_{il}=\frac{1}{\rho_{il}}\frac{\partial\rho_{il}j_{n}\left( \rho_{il} \right)}{\partial\rho_{il}}$ with the symbol $\rho_{il}=k_{i}r_{l}$, besides, $B_{\boldsymbol{M,N}}^{N1}=D_{\boldsymbol{M,N}}^{11}=0$. Rewriting the coupled recurrence Eqns. (S28a) and (S28b), we obtain the following terse form:

$$\begin{aligned} \left[ \begin{matrix} B_{\boldsymbol{M,N}}^{\left( f+1 \right)1} \\ D_{\boldsymbol{M,N}}^{\left( f+1 \right)1} \end{matrix} \right]=\left[ \begin{matrix} \frac{1}{T_{Ff}^{H,V}} & \frac{R_{Ff}^{H,V}}{T_{Ff}^{H,V}} \\ \frac{R_{Pf}^{H,V}}{T_{Pf}^{H,V}} & \frac{1}{T_{Pf}^{H,V}} \end{matrix} \right]\left[ \begin{matrix} B_{\boldsymbol{M,N}}^{f1} \\ D_{\boldsymbol{M,N}}^{f1}+\delta_{f}^{1} \end{matrix} \right],\#\left( S29 \right) \end{aligned}$$

where the symbols $T_{\left( F,P \right)f}^{H}$, $R_{\left( F,P \right)f}^{H}$ represent the transmission and reflection contributions from TE waves (corresponding to the superscript *H*), and $T_{\left( F,P \right)f}^{V}$, $R_{\left( F,P \right)f}^{V}$ represent the transmission and reflection contributions from TM waves (corresponding to the superscript *V*). The subscripts *F* and *P* denote the centrifugal and centripetal waves, respectively. Furthermore, the symbols $T_{\left( F,P \right)f}^{H,V}$, $R_{\left( F,P \right)f}^{H,V}$ can be expressed as^10^

$$\begin{aligned} T_{Ff}^{H}= \frac{\mu_{f}k_{f+1}\left( \partial j_{\left( f+1 \right)f}h_{\left( f+1 \right)f}^{\left( 1 \right)}-j_{\left( f+1 \right)f}\partial h_{\left( f+1 \right)f}^{\left( 1 \right)} \right)}{\mu_{f}k_{f+1}\partial j_{\left( f+1 \right)f}h_{ff}^{\left( 1 \right)}-\mu_{f+1}k_{f}j_{\left( f+1 \right)f}\partial h_{ff}^{\left( 1 \right)}},\#\left( S30a \right) \end{aligned}$$

$$\begin{aligned} T_{Pf}^{H}= \frac{\mu_{f}k_{f+1}\left( j_{\left( f+1 \right)f}\partial h_{\left( f+1 \right)f}^{\left( 1 \right)}-\partial j_{\left( f+1 \right)f}h_{\left( f+1 \right)f}^{\left( 1 \right)} \right)}{\mu_{f}k_{f+1}j_{ff}\partial h_{\left( f+1 \right)f}^{\left( 1 \right)}-\mu_{f+1}k_{f}\partial j_{ff}h_{\left( f+1 \right)f}^{\left( 1 \right)}},\#\left( S30b \right) \end{aligned}$$

$$\begin{aligned} T_{Ff}^{V}= \frac{\mu_{f}k_{f+1}\left( j_{\left( f+1 \right)f}\partial h_{\left( f+1 \right)f}^{\left( 1 \right)}-\partial j_{\left( f+1 \right)f}h_{\left( f+1 \right)f}^{\left( 1 \right)} \right)}{\mu_{f}k_{f+1}\partial j_{\left( f+1 \right)f}h_{ff}^{\left( 1 \right)}-\mu_{f+1}k_{f}j_{\left( f+1 \right)f}\partial h_{ff}^{\left( 1 \right)}},\#\left( S30c \right) \end{aligned}$$

$$\begin{aligned} T_{Pf}^{V}= \frac{\mu_{f}k_{f+1}\left( \partial j_{\left( f+1 \right)f}h_{\left( f+1 \right)f}^{\left( 1 \right)}-j_{\left( f+1 \right)f}\partial h_{\left( f+1 \right)f}^{\left( 1 \right)} \right)}{\mu_{f}k_{f+1}j_{ff}\partial h_{\left( f+1 \right)f}^{\left( 1 \right)}-\mu_{f+1}k_{f}\partial j_{ff}h_{\left( f+1 \right)f}^{\left( 1 \right)}},\#\left( S30d \right) \end{aligned}$$

$$\begin{aligned} R_{Ff}^{H}= \frac{\mu_{f}k_{f+1}\partial j_{\left( f+1 \right)f}j_{ff}-\mu_{f+1}k_{f}j_{\left( f+1 \right)f}\partial j_{ff}}{\mu_{f}k_{f+1}\partial j_{\left( f+1 \right)f}h_{ff}^{\left( 1 \right)}-\mu_{f+1}k_{f}j_{\left( f+1 \right)f}\partial h_{ff}^{\left( 1 \right)}},\#\left( S30e \right) \end{aligned}$$

$$\begin{aligned} R_{Pf}^{H}= \frac{\mu_{f}k_{f+1}\partial h_{\left( f+1 \right)f}^{\left( 1 \right)}h_{ff}^{\left( 1 \right)}-\mu_{f+1}k_{f}h_{\left( f+1 \right)f}^{\left( 1 \right)}\partial h_{ff}^{\left( 1 \right)}}{\mu_{f}k_{f+1}\partial h_{\left( f+1 \right)f}^{\left( 1 \right)}j_{ff}-\mu_{f+1}k_{f}h_{\left( f+1 \right)f}^{\left( 1 \right)}\partial j_{ff}},\#\left( S30f \right) \end{aligned}$$

$$\begin{aligned} R_{Ff}^{V}= \frac{\mu_{f}k_{f+1}\partial j_{ff}j_{\left( f+1 \right)f}-\mu_{f+1}k_{f}j_{ff}\partial j_{\left( f+1 \right)f}}{\mu_{f}k_{f+1}\partial h_{ff}^{\left( 1 \right)}j_{\left( f+1 \right)f}-\mu_{f+1}k_{f}h_{ff}^{\left( 1 \right)}\partial j_{\left( f+1 \right)f}},\#\left( S30g \right) \end{aligned}$$

$$\begin{aligned} R_{Pf}^{V}= \frac{\mu_{f}k_{f+1}\partial h_{ff}^{\left( 1 \right)}h_{\left( f+1 \right)f}^{\left( 1 \right)}-\mu_{f+1}k_{f}h_{ff}^{\left( 1 \right)}\partial h_{\left( f+1 \right)f}^{\left( 1 \right)}}{\mu_{f}k_{f+1}\partial j_{ff}h_{\left( f+1 \right)f}^{\left( 1 \right)}-\mu_{f+1}k_{f}j_{ff}\partial h_{\left( f+1 \right)f}^{\left( 1 \right)}}.\#\left( S30h \right) \end{aligned}$$

After solving the transmission matrix, we can finally get the scattering dyadic Green's function coefficient of the arbitrary multilayer spherical nanoparticle. Following, we need to further simplify the dyadic Green's function with the spherical harmonic addition theorem^12^

$$\begin{aligned} P_{n}\left( \cos\Theta\right)=\sum_{m=0}^{n} \left( 2-\delta_{0m} \right)\frac{\left( n-m \right)!}{\left( n+m \right)!}Y_{n}^{m}\left( \theta,\phi\right)\left[ Y_{n}^{m}\left( \theta^{'},\phi^{'} \right) \right]^{*},\#\left( S31 \right) \end{aligned}$$

where $\left( \theta,\phi\right)$ and $\left( \theta^{'},\phi^{'} \right)$ denote two different directions in our spherical polar coordinate system that are separated by an angle $\Theta$. In general, spherical trigonometry (or vector methods) shows that these angles obey the identity

$$\begin{aligned} \cos\Theta=\cos\theta\cos\theta^{'}+\sin\theta\sin\theta^{'}\cos\left( \phi-\phi^{'} \right).\#\left( S32 \right) \end{aligned}$$

In our case, the current source and response field are located at the same position $\left( \boldsymbol{r}=\boldsymbol{r}^{'} \right)$ or at the mirror position $\left( \boldsymbol{r}=-\boldsymbol{r}^{'} \right)$. Therefore, taking the spherical harmonic addition theorem with these special values, the scattering dyadic Green's function at the $s=f=1$st layer can be rewritten as^14^

$$\begin{aligned} \left. \boldsymbol{G}_{es}^{\left( 11 \right)}\left( \boldsymbol{r},\boldsymbol{r}^{'} \right) \right|_{\boldsymbol{r}=\boldsymbol{r}^{'}}&=\frac{ik_{1}}{4\pi}\sum_{eo} \sum_{n=1}^{\infty} \left( 2n+1 \right) \\ &\times\{n\left( n+1 \right)B_{\boldsymbol{N}}^{11}\left( \frac{h_{n}^{\left( 1 \right)}\left( kr \right)}{kr} \right)^{2}\hat{\boldsymbol{r}}\hat{\boldsymbol{r}} \\ &+\frac{1}{2}\left[ B_{\boldsymbol{M}}^{11}\left（ h_{n}^{\left( 1 \right)}\left( kr \right) \right）^{2}+B_{\boldsymbol{N}}^{11}\left( \frac{1}{kr}\frac{\partial rh_{n}^{\left( 1 \right)}\left( kr \right)}{\partial r} \right)^{2} \right]\hat{\boldsymbol{\theta}}\hat{\boldsymbol{\theta}} \\ &+\frac{1}{2}\left[ B_{\boldsymbol{M}}^{11}\left（ h_{n}^{\left( 1 \right)}\left( kr \right) \right）^{2}+B_{\boldsymbol{N}}^{11}\left( \frac{1}{kr}\frac{\partial rh_{n}^{\left( 1 \right)}\left( kr \right)}{\partial r} \right)^{2} \right]\hat{\boldsymbol{\phi}}\hat{\boldsymbol{\phi}}\},\#\left( S33a \right) \end{aligned}$$

$$\begin{aligned} \left. \boldsymbol{G}_{es}^{\left( 11 \right)}\left( \boldsymbol{r},\boldsymbol{r}^{'} \right) \right|_{\boldsymbol{r}=-\boldsymbol{r}^{'}}&=\frac{ik_{1}}{4\pi}\sum_{eo} \sum_{n=1}^{\infty} \left( 2n+1 \right) \\ &\times\{n\left( n+1 \right)\left( -1 \right)^{n}B_{\boldsymbol{N}}^{11}\left( \frac{h_{n}^{\left( 1 \right)}\left( kr \right)}{kr} \right)^{2}\hat{\boldsymbol{r}}\hat{\boldsymbol{r}} \\ &+\frac{1}{2}\left( -1 \right)^{n}\left[ B_{\boldsymbol{N}}^{11}\left( \frac{1}{kr}\frac{\partial rh_{n}^{\left( 1 \right)}\left( kr \right)}{\partial r} \right)^{2}-B_{\boldsymbol{M}}^{11}\left（ h_{n}^{\left( 1 \right)}\left( kr \right) \right）^{2} \right]\hat{\boldsymbol{\theta}}\hat{\boldsymbol{\theta}} \\ &+\frac{1}{2}\left( -1 \right)^{n}\left[ B_{\boldsymbol{M}}^{11}\left（ h_{n}^{\left( 1 \right)}\left( kr \right) \right）^{2}-B_{\boldsymbol{N}}^{11}\left( \frac{1}{kr}\frac{\partial rh_{n}^{\left( 1 \right)}\left( kr \right)}{\partial r} \right)^{2} \right]\hat{\boldsymbol{\phi}}\hat{\boldsymbol{\phi}}\}.\#\left( S33b \right) \end{aligned}$$

Therefore, the imaginary part of the scattering dyadic Green’s function along the $r$-direction (radial direction) is

$$\begin{aligned} \mathrm{Im} \left. \boldsymbol{G}_{es}^{\left( 11 \right)}\left( \boldsymbol{r},\boldsymbol{r}^{'} \right) \right|_{rr}=\frac{k_{1}}{4\pi} \mathrm{Re} \left\{ \begin{matrix} \sum_{n=1}^{\infty} n\left( n+1 \right)\left( 2n+1 \right)B_{\boldsymbol{N}}^{11}\left( \frac{h_{n}^{\left( 1 \right)}\left( kr \right)}{kr} \right)^{2}, \left( \boldsymbol{r}=\boldsymbol{r}^{'} \right) \\ \sum_{n=1}^{\infty} n\left( n+1 \right)\left( 2n+1 \right)\left( -1 \right)^{n}B_{\boldsymbol{N}}^{11}\left( \frac{h_{n}^{\left( 1 \right)}\left( kr \right)}{kr} \right)^{2},\left( \boldsymbol{r}=-\boldsymbol{r}^{'} \right) \end{matrix} \right..\#\left( S34 \right) \end{aligned}$$

Using a similar method, we can obtain the imaginary part of the dyadic Green's function $\left( \boldsymbol{r}=\boldsymbol{r}^{'} \right)$ along the transition direction of the emitter in a homogeneous medium as^14^

$$\begin{aligned} \mathrm{Im}\boldsymbol{G}_{e0}&=\frac{k_{1}}{4\pi} \sum_{n=1}^{\infty} n\left( n+1 \right)\left( 2n+1 \right)\left( \frac{j_{n}\left( kr \right)}{kr} \right)^{2} \\ &=\frac{k_{1}}{6\pi}.\#\left( S35 \right) \end{aligned}$$

We have successfully obtained the simplified form and boundary coefficients of the dyadic Green's function. Meanwhile, we obtain the analytical solution of the imaginary part and apply it for calculating LDOS.

1. Details on Deep Learning and Reverse Design Algorithm

In this work, deep learning (DL) is applied to a specific quantum nanophotonic system that is QEs near a multilayer shell-metal-nanoparticle (SMNP). Here, we take the full-connected neural network (NN) with 4 hidden layers and 1600 neurons in each segment. The input is the shell thicknesses, and the output is the enhancement of the local-density-of-states (LDOS). The NN was trained with L2 regularization and RMSprop algorithm. Moreover, the hyperparameters for NNs training are determined by experience, and the main hyperparameters are shown in Tbl. S1.

**Table S1: The hyperparameters for neural networks (NNs) training.**

| **Hyperparameter** | **Value** |
| --- | --- |
| Batch Size | 100 |
| Patience Limit | 10 |
| Learning Rate (LR) | 0.05 |
| Decay of LR | 0.995 |
| L2 Regularization | 0.0001 |
| Normalization | 0 (bool) |

The hyperparameters include the essential hyperparameters (batch size, patience limit, learning rate (LR) and decay of LR) and optional hyperparameters (L2 Regularization and Normalization).

With the hyperparameters, we successfully train NNs map to different multilayer (2-layer, 4-layer, 6-layer, and 8-layer) SMNPs and the enhancement of LDOS. The enhancement of LDOS can be solved with the Maxwell equations^10^. Here, we prepare the datasets with 5×10^5^ samples, and each dataset is split into 0.8 for training and 0.2 for verification. The performance of NNs is displayed by the accuracy test, where we take the mean relative accuracy (MRA). The MRA is presented as the mean off per point on the enhancement of LDOS. And the accuracy results are shown in Tbl. S2.

**Table S2: Accuracy results for various multilayer shell-metal-nanoparticles (SMNPs)**

| **Shells** | **MRA (training)** | **MRA (validation)** |
| --- | --- | --- |
| 2 | 99.27% | 99.32% |
| 4 | 92.39% | 91.73% |
| 6 | 87.83% | 86.46% |
| 8 | 85.08% | 84.02% |

The mean relative accuracy (MRA) represents the average of the output value. The MRA of training and validation are close inferring that not much overfitting is occurring.

The outcomes convey that the NNs of different multilayer SMNPs perform excellently in fitting the enhancement of LDOS. Comparing the MRA of different multilayer SMNPs, as the number of layers increases, the accuracy drops rapidly. Because the solution range increases exponentially with the number of layers. Nevertheless, the accuracy rate of the 8-layer SMNP is as high as 85%, since the dataset with enough samples. Moreover, we take these NNs to fit the enhancement of LDOS in the validation datasets, and the results as shown in Fig. S1.

The results show that NNs can fit the enhancement of LDOS with different multilayer SMNPs, but the errors are increased rapidly as the shell grow in number, which is same as the MRA results. Specially, the peak values are difficult to estimate, since the value range is wide from 0 to 1×10^5^. Nevertheless, the NNs also can be applied to inverse design the shell thicknesses of SMNPs. Next, we take the back-propagation algorithm as the inverse design algorithm and achieve it with Python and Pytorch.


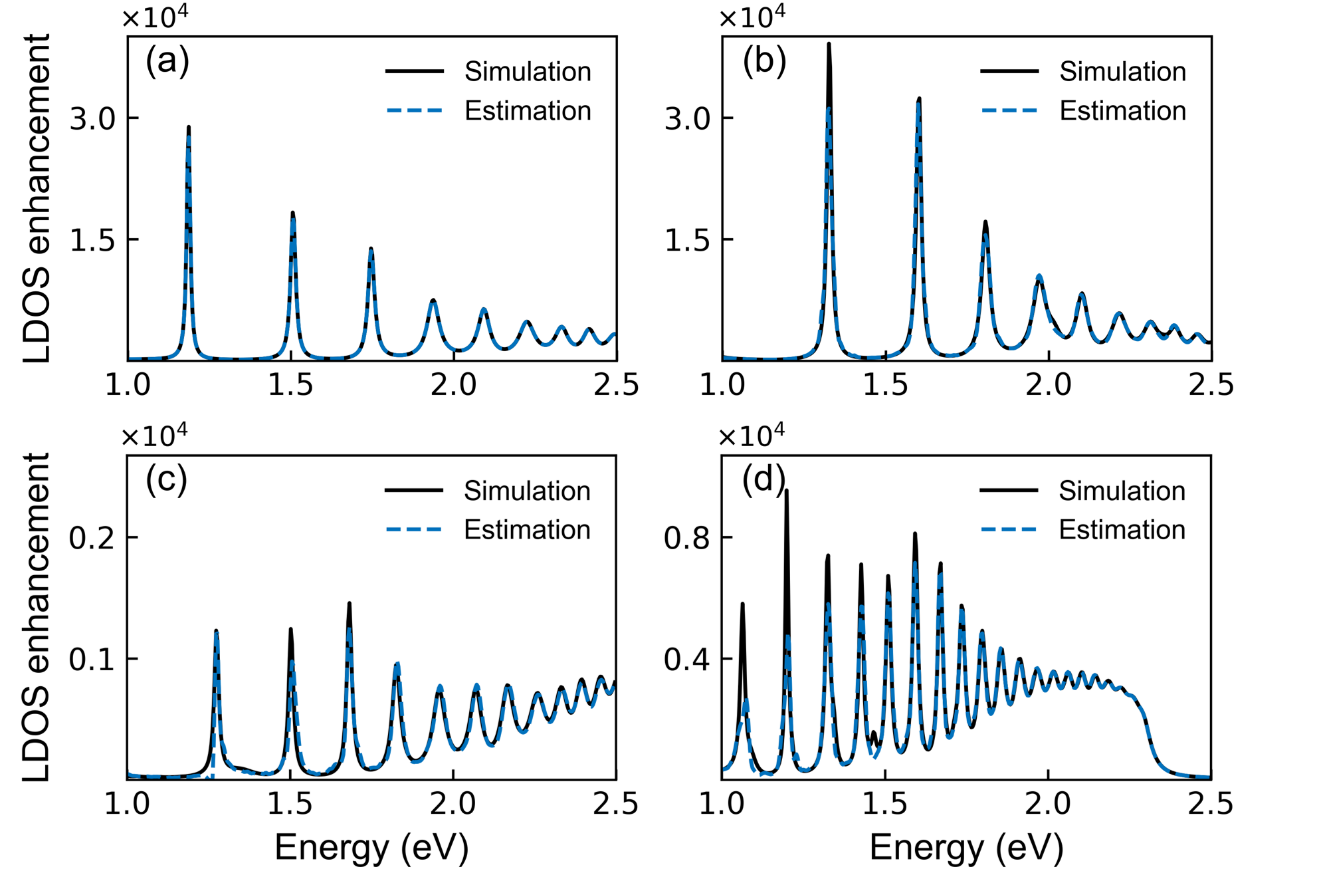


**Figure S1: Fitting examples of NNs with multilayer SMNPs.** The results of the multilayer (2-layer, 4-layer, 6-layer, and 8-layer) SMNPs are plotted in (a), (b), (c), and (d), respectively, with simulation spectra (black lines) in the validation datasets, and NNs estimation results (blue lines).

Pytorch (python library of deep learning) is the preferred framework to program the DL, which provides easy modules and operates flexibly. We employ the back-propagation algorithm to optimize our input variables^15^. However, the library without any optimize or design module, so that we considered a tandem network structure to implement the reverse design algorithm (Fig. S2). The structure not only makes us can tuning the input parameters but also can consider the boundary of the input parameters. It makes DL can be used in different situations with several boundaries and conditions.


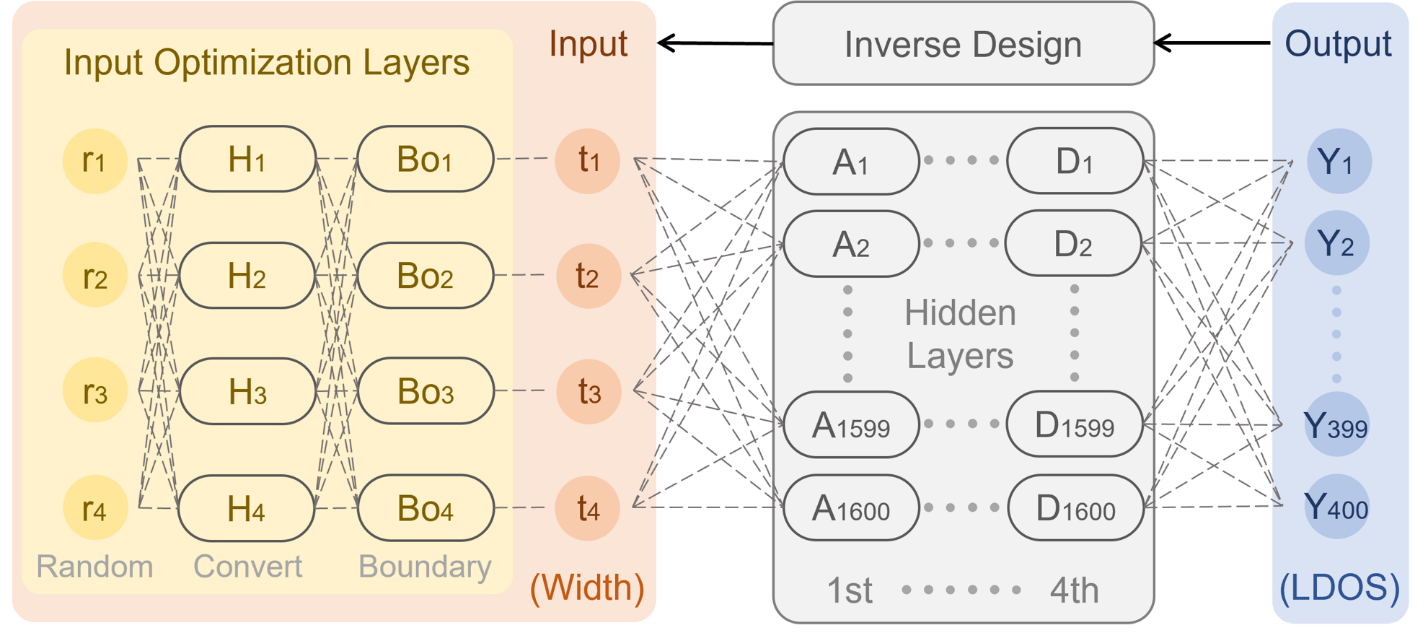


**Figure S2: Structure of a tandem network to comply the reverse design algorithm.** A layer of full connection and a layer of boundary conditions are added in front of the full-connected network.

1. Coupling Strength Optimization for Entanglement Dynamics


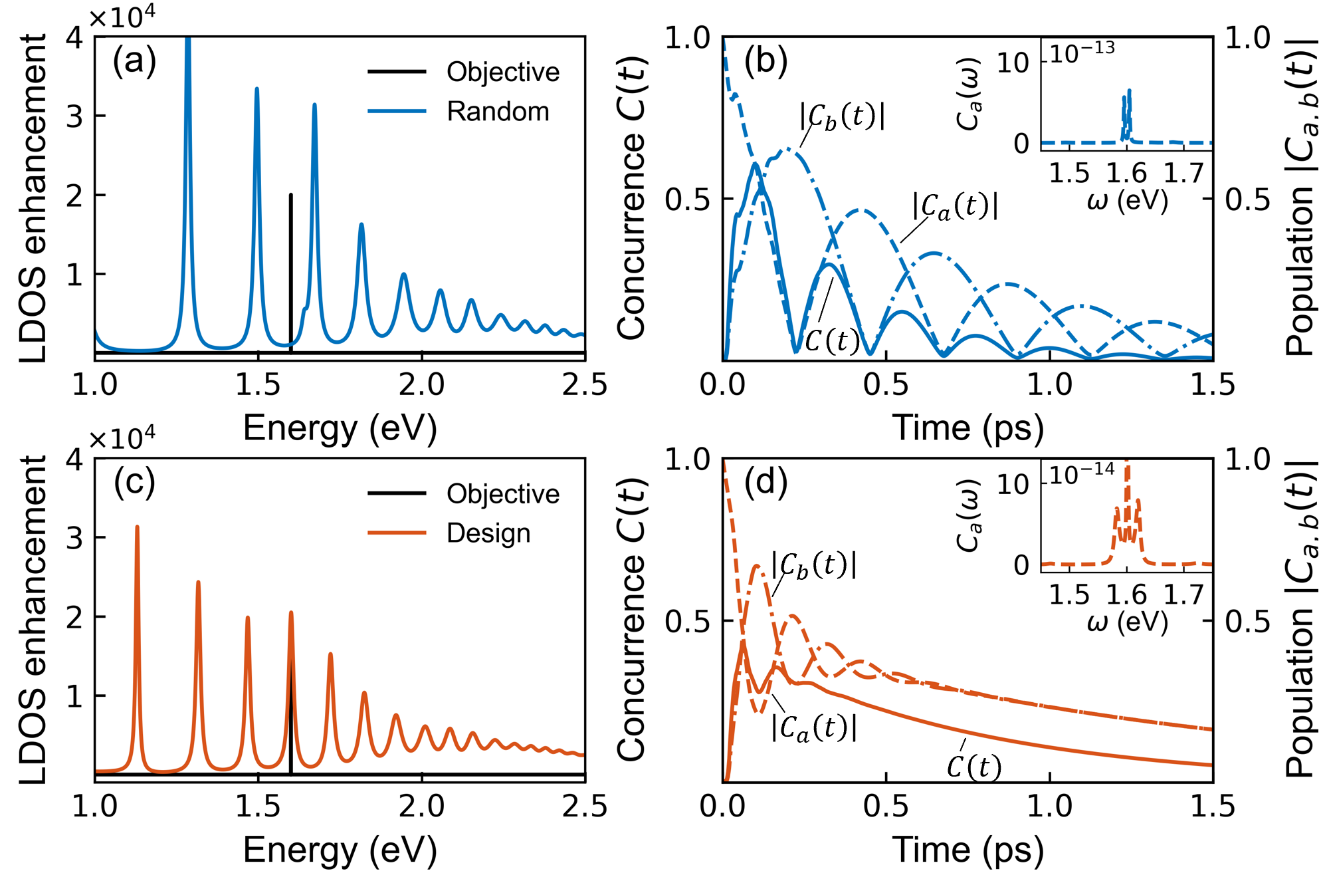


**Figure S3: Optimizing the coupling strength of entanglement dynamics in the quantum nanophotonic systems consisting of a 4-layer SMNP and two QEs (**$\mathbf{QE}_{\boldsymbol{a}}$ **and** $\mathbf{QE}_{\boldsymbol{b}}$**).** Comparison between unoptimized random structure ($t_{1}=1.02 \mathrm{nm}$, $t_{2}=11.20 \mathrm{nm}$, $t_{3}=1.52 \mathrm{nm}$, $t_{4}=4.40 \mathrm{nm}$) (**upper**) and optimized design structure ($t_{1}=1.21 \mathrm{nm}$, $t_{2}=20.84 \mathrm{nm}$, $t_{3}=2.20 \mathrm{nm}$, $t_{4}=4.18 \mathrm{nm}$) (**lower**) to achieve strong coupling between the SMNP and the QEs. (a) and (c) plot the enhancement of LDOS corresponding to the 4-layer SMNP. (b) and (d) plot the Concurrence $C(t)$ (solid line) and population $|C_{a,b}(t)|$ (dashed lines) and its spectrum of $\mathrm{QE}_{a}$ $C_{a}(\omega)$ (Inset).

The quantum nanophotonic system can form entanglement between a pair of QEs. Here, we display the effect of the amplitude of the LDOS enhancement on the entanglement dynamics. We obtain the entanglement dynamics results of the 4-layer SMNPs as shown in Fig. S3. The upper panel (Figs. S3a and S3b) shows the results of a random SMNP structure without optimization, whereas the lower panel (Figs. S3c and S3d) demonstrates the results of an optimized design SMNP. The difference between these two LDOS enhancements (Figs. S3a and S3c) is the detuning of the resonant frequency in the random SMNP, while the amplitude of the LDOS enhancement is higher in the design SMNP.

To quantify the quantum entanglement between the two QEs, the Concurrence is often used and defined as $C(t) = 2\max\left[ 0, \left| C_{a}\left( t \right)C_{b}^{*}(t) \right| \right]$, ranging from zero for separable states up to 1 for maximally entangled states, where the oscillation period characterizes the coupling strength of the entanglement and the slope of $C(t)$ represents the decay rate of the entanglement^16^. Together with the population dynamics $\left| C_{a,b}(t) \right|$ of the two QEs, we plot the Concurrence dynamics $C\left( t \right)$ in Figs. S3b and S3d, where the corresponding spectrum $C_{a}\left( \omega\right)$ for one of QEs are drawn in the Insets. Firstly, the Concurrence and the population for the random SMNP (Fig. S3b) obviously generate a larger oscillation period, while the curves for the design SMNP with have a smaller oscillation period. Besides, the spectra $C_{a}\left( \omega\right)$ (Inset) display the Rabi splitting, in which the random SMNP has a small Rabi frequency (Fig. S3d) and the design SMNP has a large Rabi frequency (Fig. S3b). These features both prove that the coupling strength of the entanglement are optimized by the amplitude of the LDOS enhancement. To obtain the strong entanglement, it is required a relative high amplitude of the LDOS enhancement.

1. Decay Rate Optimization for Entanglement Dynamics with the Lorentz Function Objective


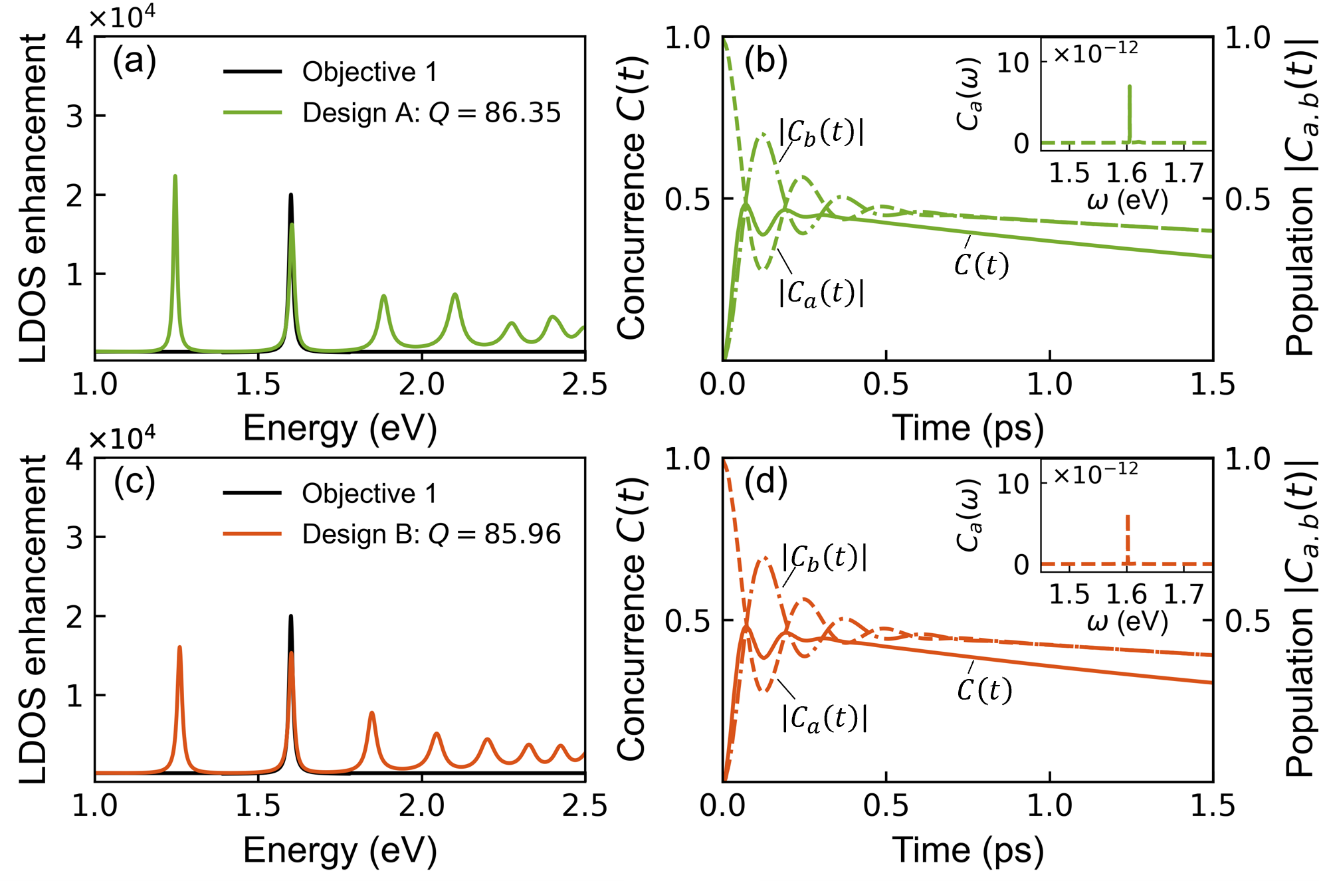


**Figure S4:** **Optimizing the decay rate of entanglement dynamics in the quantum nanophotonic systems consisting of a 4-layer or 2-layer SMNP and two QEs (**$\mathbf{QE}_{\boldsymbol{a}}$ **and** $\mathbf{QE}_{\boldsymbol{b}}$**).** (a) plot the enhancement of LDOS corresponding to the 4-layer SMNP ($t_{1}=1.75 \mathrm{nm}$, $t_{2}=4.69 \mathrm{nm}$, $t_{3}=3.72 \mathrm{nm}$, $t_{4}=9.11 \mathrm{nm}$ with the Q-factor $Q=86.35$). (c) plot the enhancement of LDOS corresponding to the 2-layer SMNP ($t_{1}=2.11 \mathrm{nm}$, $t_{2}=21.61 \mathrm{nm}$, with the Q-factor $Q=85.96$). (b) and (d) plot the Concurrence $C(t)$ (solid line) and population $|C_{a,b}(t)|$ (dashed lines) and its spectrum of $\mathrm{QE}_{a}$ $C_{a}(\omega)$ (Inset).

Here, we display another method to optimize the decay rate of the entanglement dynamics. The method is using the Lorentz function, which have been utilized to design the Q-factor^17^. It is also easy to optimize the LDOS enhancement to be stable. We obtain two optimized entanglement dynamics results of the 4-layer and 2-layer SMNPs as shown in Fig. S4.

The figures show that the second method can effectively design the entanglement dynamics of the quantum nanophotonic systems consisting of a 4-layer or 2-layer SMNP and two QEs ($\mathrm{QE}_{a}$ and $\mathrm{QE}_{b}$). The Q-factors of these results are over 85. The Concurrence $C(t)$ (solid line) and population $|C_{a,b}(t)|$ (dashed lines) are stable, and the populations are near 0.5, with spectrum $C_{a}(\omega)$ of $\mathrm{QE}_{a}$ has a high peak closed to 1×10^-11^. Therefore, this method also is an effectively way to optimize the decay rate of entanglement dynamics with Q-factor, and it can be used in other quantum nanophotonic systems with different-layer SMNP.

1. Multiple Optimizations with Partially Fixed Thicknesses


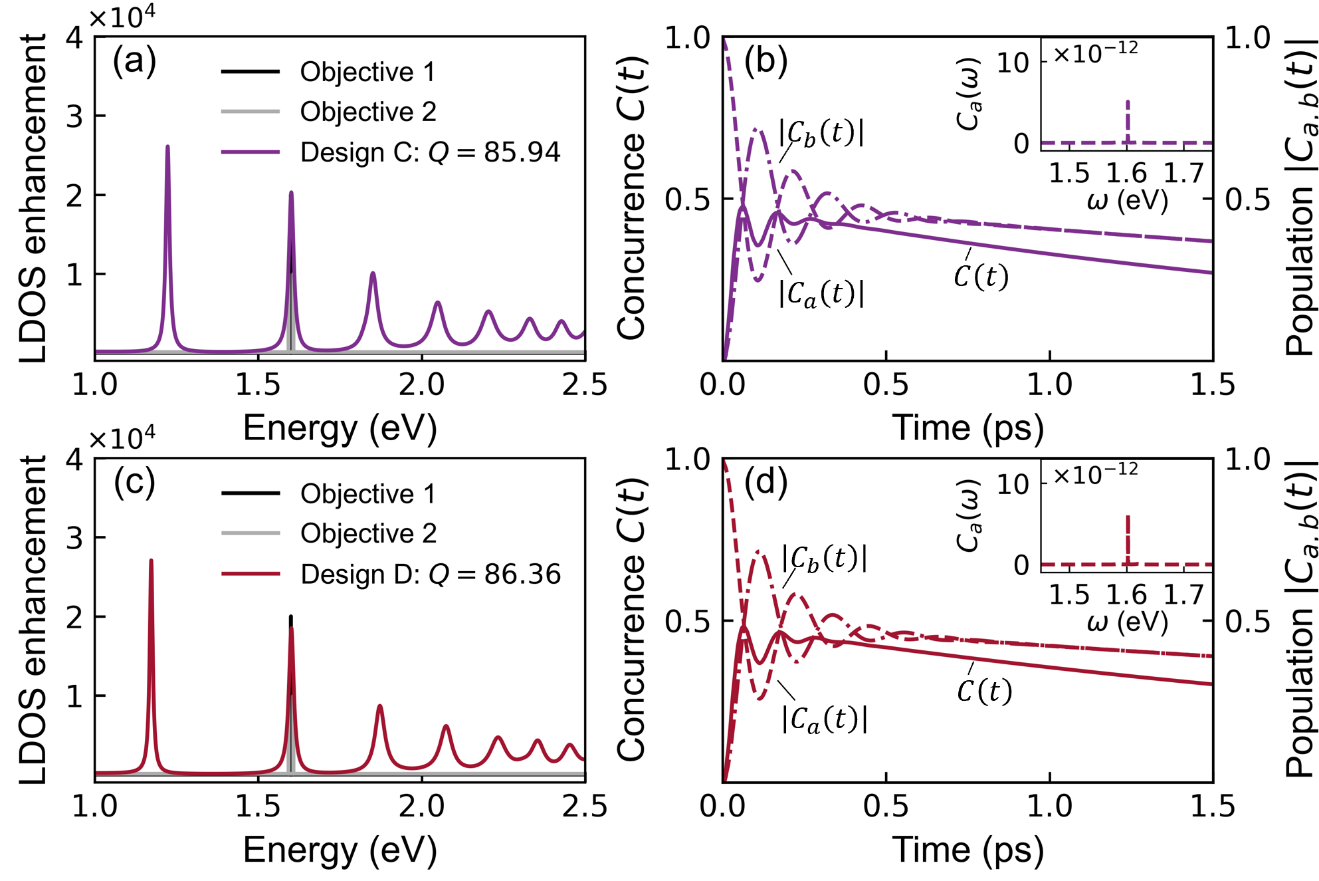


**Figure S5**: **Multiple optimizations with partially fixed parameters (**$\boldsymbol{t}_{\boldsymbol{4}}\boldsymbol{=3.45}\mathbf{nm}$ **or** $\boldsymbol{t}_{\boldsymbol{3}}\boldsymbol{=4.65}\mathbf{nm}$**,** $\boldsymbol{t}_{\boldsymbol{4}}\boldsymbol{=3.45}\mathbf{nm}$**) in the quantum nanophotonic systems consisting of a 4-layer and two QEs (**$\mathbf{QE}_{\boldsymbol{a}}$ **and** $\mathbf{QE}_{\boldsymbol{b}}$**).** (a) plot the enhancement of LDOS corresponding to the 4-layer SMNP with one fixed parameter ($t_{1}=1.83 \mathrm{nm}$, $t_{2}=13.52 \mathrm{nm}$, $t_{3}=1.72 \mathrm{nm}$, $t_{4}=3.45 \mathrm{nm}$ with the Q-factor $Q=85.94$). (c) plot the enhancement of LDOS corresponding to the 4-layer SMNP with two fixed parameters ($t_{1}=1.93 \mathrm{nm}$, $t_{2}=10.91 \mathrm{nm}$, $t_{3}=4.65 \mathrm{nm}$, $t_{4}=3.45 \mathrm{nm}$, with the Q-factor $Q=86.36$). (b) and (d) plot the Concurrence $C(t)$ (solid line) and population $|C_{a,b}(t)|$ (dashed lines) and its spectrum of $\mathrm{QE}_{a}$ $C_{a}(\omega)$ (Inset).

Here, we display an advantage of the back-propagation algorithm to optimize the entanglement dynamics with partially fixed parameters. Based on the designed structure ($t_{1}=1.84 \mathrm{nm}$, $t_{2}=3.73 \mathrm{nm}$, $t_{3}=4.65 \mathrm{nm}$, $t_{4}=3.45 \mathrm{nm}$), we fix the core thickness ($t_{4}=3.45 \mathrm{nm}$) or the core and a shell thicknesses ($t_{3}=4.65 \mathrm{nm}$, $t_{4}=3.45 \mathrm{nm}$) to optimize the entanglement dynamics. We obtain two optimized results with different thicknesses of the 4-layer SMNPs as shown in Fig. S5. The figures show that we can fix partial parameters to inverse design the other parameter to meet our expected objective. Although the specific layer thicknesses are different, the LDOS enhancements (Figs. S5a and S5c) are basically consistent. Besides the Q-factors of these results are over 85. The Concurrence $C(t)$ (solid line) and population $|C_{a,b}(t)|$ (dashed lines) are stable (Figs. S5b and S5d), and the populations are near 0.5, with spectrum $C_{a}(\omega)$ of $\mathrm{QE}_{a}$ has a high peak closed to 1×10^-11^. Therefore, with the diverse inverse design structures, the inverse design framework can be used in various applications and in actual experiments.

**Reference**

(1) Vogel, W.; Welsch, D. *Quantum Optics*, 1st ed.; Wiley, 2006.

(2) Peřina, J. *Coherence and Statistics of Photons and Atoms*; Wiley series in lasers and applications; Wiley: New York, 2001.

(3) Knoll, L.; Scheel, S.; Welsch, D.-G. QED in Dispersing and Absorbing Media. arXiv June 26, 2003.

(4) Dung, H. T.; Knöll, L.; Welsch, D.-G. Three-Dimensional Quantization of the Electromagnetic Field in Dispersive and Absorbing Inhomogeneous Dielectrics. *Phys. Rev. A* **1998**, *57* (5), 3931–3942.

(5) Khanbekyan, M.; Wiersig, J. Decay Suppression of Spontaneous Emission of a Single Emitter in a High- Q Cavity at Exceptional Points. *Phys. Rev. Research* **2020**, *2* (2), 023375.

(6) Wubs, M.; Suttorp, L. G.; Lagendijk, A. Multiple-Scattering Approach to Interatomic Interactions and Superradiance in Inhomogeneous Dielectrics. *Phys. Rev. A* **2004**, *70* (5), 053823.

(7) Chen, G.; Yu, Y.-C.; Zhuo, X.-L.; Huang, Y.-G.; Jiang, H.; Liu, J.-F.; Jin, C.-J.; Wang, X.-H. Ab Initio Determination of Local Coupling Interaction in Arbitrary Nanostructures: Application to Photonic Crystal Slabs and Cavities. *Phys. Rev. B* **2013**, *87* (19), 195138.

(8) Liu, J.; Chen, G.; Li, L.; Liu, R.; Li, W.; Liu, G.; Wu, F.; Chen, Y. Radiative Coupling of Two Quantum Emitters in Arbitrary Metallic Nanostructures. *Sci Rep* **2022**, *12* (1), 6901.

(9) Dzsotjan, D.; Kästel, J.; Fleischhauer, M. Dipole-Dipole Shift of Quantum Emitters Coupled to Surface Plasmons of a Nanowire. *Phys. Rev. B* **2011**, *84* (7), 075419.

(10) Le-Wei, L; Pang-Shyan, K; Mook-Seng, L; Tat-Soon, Y. Electromagnetic Dyadic Green’s Function in Spherically Multilayered Media. *IEEE Trans. Microwave Theory Techn.* **1994**, *42* (12), 2302–2310.

(11) Tai, C. *Dyadic Green Functions in Electromagnetic Theory*, 2nd ed.; IEEE Press series on electromagnetic waves; IEEE Press: Piscataway, NJ, 1994.

(12) Riley, K. F.; Hobson, M. P.; Bence, S. J. *Mathematical Methods for Physics and Engineering*, 3rd ed.; Cambridge University Press: Cambridge ; New York, 2006.

(13) Ali, S. M.; Habashy, T. M.; Kong, J. A. Spectral-Domain Dyadic Green’s Function in Layered Chiral Media. *J. Opt. Soc. Am. A, JOSAA* **1992**, *9* (3), 413–423.

(14) Hakami, J.; Wang, L.; Zubairy, M. S. Spectral Properties of a Strongly Coupled Quantum-Dot--Metal-Nanoparticle System. *Phys. Rev. A* **2014**, *89* (5), 053835.

(15) Peurifoy, J.; Shen, Y.; Jing, L.; Yang, Y.; Cano-Renteria, F.; DeLacy, B. G.; Joannopoulos, J. D.; Tegmark, M.; Solja, M. Nanophotonic Particle Simulation and Inverse Design Using Artificial Neural Networks. *Sci. Adv.* **2018**, 8.

(16) Maniscalco, S.; Francica, F.; Zaffino, R. L.; Lo Gullo, N.; Plastina, F. Protecting Entanglement via the Quantum Zeno Effect. *Phys. Rev. Lett.* **2008**, *100* (9), 090503.

(17) Xu, L.; Rahmani, M.; Ma, Y.; Smirnova, D. A.; Kamali, K. Z.; Deng, F.; Chiang, Y. K.; Huang, L.; Zhang, H.; Gould, S.; Neshev, D. N.; Miroshnichenko, A. E. Enhanced Light–Matter Interactions in Dielectric Nanostructures via Machine-Learning Approach. *Adv. Photon.* **2020**, *2* (02), 1.
